# Changes in QTc Interval in the Citalopram for Agitation in Alzheimer's Disease (CitAD) Randomized Trial

**DOI:** 10.1371/journal.pone.0098426

**Published:** 2014-06-10

**Authors:** Lea T. Drye, David Spragg, D. P. Devanand, Constantine Frangakis, Christopher Marano, Curtis L. Meinert, Jacobo E. Mintzer, Cynthia A. Munro, Gregory Pelton, Bruce G. Pollock, Anton P. Porsteinsson, Peter V. Rabins, Paul B. Rosenberg, Lon S. Schneider, David M. Shade, Daniel Weintraub, Jerome Yesavage, Constantine G. Lyketsos, for the CitAD Research Group

**Affiliations:** 1 Department of Epidemiology and Center for Clinical Trials, Johns Hopkins Bloomberg School of Public Health, Baltimore, Maryland, United States of America; 2 Electrophysiology Laboratory, Johns Hopkins Hospital and Johns Hopkins Bayview Medical Center, Baltimore, Maryland, United States of America; 3 Division of Geriatric Psychiatry, College of Physicians and Surgeons, Columbia University, New York, New York, United States of America; 4 Department of Biostatistics, Johns Hopkins Bloomberg School of Public Health, Baltimore, Maryland, United States of America; 5 Division of Geriatric Psychiatry and Neuropsychiatry, Johns Hopkins University School of Medicine, Johns Hopkins Bayview Medical Center, Baltimore, Maryland, United States of America; 6 Departments of Epidemiology and Biostatistics, Johns Hopkins Bloomberg School of Public Health, Baltimore, Maryland, United States of America; 7 Clinical Biotechnology Research Institute, Roper St. Francis Healthcare, Department of Health Studies, Medical University of South Carolina, Ralph H. Johnson VA Medical Center, Charleston, South Carolina, United States of America; 8 Departments of Psychiatry and Behavioral Sciences and Neurology, Johns Hopkins Bayview and Johns Hopkins University School of Medicine, Baltimore, Maryland, United States of America; 9 Division of Geriatric Psychiatry, New York State Psychiatric Institute, College of Physicians and Surgeons of Columbia University, New York, New York, United States of America; 10 Campbell Family Mental Health Research Institute, Division of Geriatric Psychiatry and Centre for Addiction and Mental Health, University of Toronto, Toronto, Ontario, Canada; 11 Alzheimer's Disease Care, Research and Education Program (AD-CARE), University of Rochester School of Medicine and Dentistry, Rochester, New York, United States of America; 12 Department of Psychiatry and Behavioral Sciences, Johns Hopkins University School of Medicine and Johns Hopkins Hospital, Baltimore, Maryland, United States of America; 13 Department of Psychiatry and Behavioral Sciences, John Hopkins University School of Medicine and Johns Hopkins Bayview Medical Center, Baltimore, Maryland, United States of America; 14 Departments of Psychiatry, Neurology, and Gerontology, Keck School of Medicine, University of Southern California, Los Angeles, California, United States of America; 15 Departments of Medicine (Pulmonary) and Epidemiology and Center for Clinical Trials, Johns Hopkins Bloomberg School of Public Health, Baltimore, Maryland, United States of America; 16 Departments of Psychiatry and Neurology, Perelman School of Medicine at the University of Pennsylvania, Parkinson's Disease Research, Education and Clinical Center (PADRECC), Mental Illness Research, Education and Clinical Center (MIRECC), Philadelphia Veterans Affairs Medical Center, Philadelphia, Pennsylvania, United States of America; 17 Aging Clinical Research Center, Mental Illness Research Education and Clinical Center, Veterans Affairs Palo Alto Health Care System, Departments of Psychiatry and Behavioral Sciences, Stanford University School of Medicine, Palo Alto, California, United States of America; 18 Memory and Alzheimer's Treatment Center, Department of Psychiatry and Behavioral Sciences, Johns Hopkins Bayview and Johns Hopkins School of Medicine, Baltimore, Maryland, United States of America; Glaxo Smith Kline, Denmark

## Abstract

**Background:**

A Food and Drug Administration (FDA) safety communication in August 2011 warned that citalopram was associated with a dose dependent risk of QT prolongation and recommended dose restriction in patients over the age of 60 but did not provide data for this age group.

**Methods:**

CitAD was a randomized, double-masked, placebo-controlled, multicenter clinical trial for agitation in Alzheimer's disease (AD). Participants were assigned to citalopram (target dose of 30 mg/day) or placebo in a 1∶1 ratio. 186 people, 181 of whom were over the age of 60, having probable AD with clinically significant agitation were recruited from September 2009 to January 2013. After the FDA safety communication about citalopram, ECG was added to the required study procedures before enrollment and repeated at week 3 to monitor change in QTc interval. Forty-eight participants were enrolled after enhanced monitoring began.

**Results:**

Citalopram treatment was associated with a larger increase in QTc interval than placebo (difference in week 3 QTc adjusting for baseline QTc: 18.1 ms [95% CI: 6.1, 30.1]; p = 0.004). More participants in the citalopram group had an increase ≥30 ms from baseline to week 3 (7 in citalopram versus 1 in placebo; Fisher's exact p = 0.046), but only slightly more in the citalopram group met a gender-specific threshold for prolonged QTc (450 ms for males; 470 ms for females) at any point during follow-up (3 in citalopram versus 1 in placebo, Fisher's exact p = 0.611). One of the citalopram participants who developed prolonged QTc also displayed ventricular bigeminy. No participants in either group had a cardiovascular-related death.

**Conclusion:**

Citalopram at 30 mg/day was associated with improvement in agitation in patients with AD but was also associated with QT prolongation.

**Trial Registration:**

ClinicalTrials.gov NCT00898807

## Introduction

The QT interval is measured on an electrocardiogram (ECG) as the time from the beginning of the QRS complex to the end of the T wave and corresponds to the time from onset of ventricular depolarization to completion of repolarization. The QT interval shortens as heart rate increases. The QTc interval is a standardization of the QT interval correcting for heart rate. Many formulae have been developed for calculating the QTc value and there is no consensus on the correct conversion [1], [2] but most formulae provide similar results for diagnosis of QT prolongation with heart rates in the range of approximately 60–90 beats/minute [3]. Extreme QTc prolongation (QTc>500 milliseconds) can result in electrical instability during repolarization, increasing the risk of polymorphic ventricular tachycardia (*Torsade de pointes*; TdP) and sudden cardiac death [4], [5]. A review of the literature found consistent associations between prolonged QTc and death in high risk populations [6] and a meta-analysis of observational data found that the pooled estimates support associations between prolonged QTc and death in the general population [7]. Although QT prolongation can be caused by heritable disorders in cardiac ion channel expression, numerous marketed drugs are also known to lengthen the QT interval (QTdrugs.org) in people without genetic abnormalities.

Citalopram is a selective serotonin reuptake inhibitor (SSRI) antidepressant that is frequently used in older adults [8], [9]. On 24 August 2011, the US Food and Drug Administration (FDA) issued a Drug Safety Communication [10] regarding the dose dependent risk of QT prolongation with citalopram. The communication was issued because of post-marketing reports of QT prolongation and TdP and results of an unpublished, randomized, controlled, multi-center, double-blind, crossover study that enrolled 119 healthy, non-depressed adults. Participants in the FDA QT study received citalopram 20 mg/day and 60 mg/day. The mean change in QTc interval was 8.5 milliseconds (ms) for 20 mg/day and 18.5 ms for 60 mg/day [10]. The FDA QT study included adults aged 19 to 45 (Thomas P Laughren, personal communication). The 2011 FDA communication stated that 20 mg per day was the maximum recommended dose for patients greater than 60 years of age [10], but this language was not changed on the FDA label for Celexa (brand name for citalopram) at that time. The Pharmacovigilance Working Party of the European Medicines Agency also recommended the same changes to maximum dose in summaries of product characteristics and package leaflets of citalopram products in the EU [11] and these were accepted by the co-ordination Group for Mutual Recognition and Decentralised Procedures (CMDh) agency in October 2011 [12]. The Medicines and Healthcare Products Regulatory Agency (MHRA) in the UK issued a similar advisory regarding citalopram use in December 2011 [13].

Seven months later, on 28 March 2012, the FDA issued another Drug Safety Communication and again updated the citalopram labeling [14]. The revised warning recommended that citalopram not be used by patients who have one or more of the following conditions: congenital long QT syndrome, bradycardia, hypokalemia, hypomagnesemia, recent acute myocardial infarction, or uncompensated heart failure. This 2012 communication accompanied new changes to the Celexa label, and recommended a maximum dose of 20 mg in individuals over age 60.

Following the FDA warnings, an observational study of data from a large New England healthcare setting also found dose-response associations between citalopram, escitalopram and amitriptyline with QTc prolongation but no associations for other antidepressants examined including other SSRIs fluoxetine, paroxetine and sertraline as well as other antidepressants duloxetine, venlafaxine, buproprion, mirtazapine and nortriptyline. The New England study included adults ages ≥18 and the mean age was 58 (SD = 16) years [15].

In contrast, an observational study of data from the Veterans Health Administration (VHA) National Registry for Depression found no increased risk of ventricular arrhythmia or mortality with citalopram doses of greater than 40 mg/day. 41% of the VHA cohort was age ≥60 [16]. No QTc data were presented.

To the best of our knowledge, no other trials have published on the relationship between citalopram and QT interval in older adults participating in a placebo-controlled trial. The Citalopram for agitation in Alzheimer's Disease (CitAD) trial was designed to evaluate the efficacy of citalopram for the treatment of clinically significant agitation in patients with AD. Following the FDA safety warnings regarding citalopram, CitAD investigators added safety monitoring for QT prolongation and the objective here is to show the differences in QT interval for citalopram versus placebo in older adults with AD.

## Methods

### CitAD design

CitAD was an investigator-initiated clinical trial funded by the National Institute on Aging (NIA) and National Institute of Mental Health (NIMH) than enrolled participants from August 2009 to January 2013. CitAD is registered at clinicaltrials.gov: NCT00898807. The study had eight recruiting clinical centers (seven in the U.S. and one in Canada) and two resource centers (the chair's office and the coordinating center). Study participants were recruited from memory clinics, geriatric psychiatry clinics, Veterans Administration geriatric clinics, nursing homes, community outreach, and Alzheimer Research Centers. CitAD participants had probable Alzheimer's disease as defined by NINCDS-ADRDA [17] criteria, Mini-Mental State Examination (MMSE) [18] scores of 5–28 inclusive, and clinically significant agitation. The detailed list of eligibility criteria for CitAD has been published previously [19]. The protocol for CitAD and supporting CONSORT checklist are available as supporting information; see Protocol S1 and Checklist S1.

Participants were randomized using a plan developed by the coordinating center in a 1∶1 ratio to receive citalopram or matching placebo in parallel. The treatment assignment was given in form of a medication kit identification code only after eligibility was confirmed (allocation concealment). A detailed description of the randomization and concealment methods has been published [19]. A starting dose of 10 mg was titrated up over 2 weeks to the target of 30 mg daily, provided as a single dose in the morning of three capsules each containing 10 mg. Citalopram was purchased on from commercial wholesale drug suppliers on the open market and over-encapsulated to facilitate participant and study staff masking.

Participants were followed via in-person study visits at weeks 3, 6, and 9 following enrollment and telephone contacts at weeks 1, 2, 4.5 and 7.5 weeks following enrollment. The data collection schedule was described in detail previously [19]. Adverse events were primarily collected via systematic, close-ended questions regarding known or expected side effects or complications of citalopram, and through the measurement of electrolyte levels. Serious adverse events were collected on an individual basis at the time of the event and when updated information became available.

The primary results of the CitAD trial have been reported [20] and in short, citalopram (at target does of 30 mg/day) improved agitation, global function, and caregiver burden but was associated with cognitive worsening and increased anorexia, diarrhea, fever and falls.

### Ethics statement

The study protocol was reviewed and approved by the ethics committee at each clinical center and the coordinating center: New York State Psychiatric Institute Institutional Review Board (Columbia), University Of Pennsylvania Office Of Regulatory Affairs Institute Institutional Review Board, Centre For Addiction And Mental Health Research Ethics Board (Toronto), Stanford University Panel On Human Subjects In Medical Research, University Of Southern California Keck School Of Medicine Affairs Institute Institutional Review Board, Medical University of South Carolina Institute Institutional Review Board For Human Research, Johns Hopkins Office Of Human Subject Research Institute Institutional Review Boards, Johns Hopkins Bloomberg School of Public Health Institutional Review Board, University of Rochester Research Subjects Review Board.

Participants provided written consent if they were capable of providing consent. If the participant is not fully capable of providing consent, then written consent was obtained from an authorized legal representative and the participant was asked to provide assent.

### QTc monitoring

In September 2011, the CitAD Steering Committee (SC) decided that active subjects should be informed about the FDA advisory and that participants on 3 study capsules per day (corresponding to 30 mg daily for those in the citalopram group) have an ECG as soon as was practical. New dose increases over 20 mg were temporarily halted while the SC waited for the updated Celexa label to be released.

When the changed label became available shortly after the first safety communication, there was no change regarding the use of the medication in individuals over age 60. As a result, the SC concluded that no change in the dosing scheme was needed but added an exclusion criterion for individuals with a prolonged QTc, defined as >450 ms for men and >470 ms for women, as measured on an ECG conducted at the study's screening/enrollment visit using equipment available at the clinical centers; there was no central calibration of machines or central reading facility. The cutoffs for prolonged QTc were based on recommendations from an SC-appointed study cardiologist and are consistent with standard thresholds [4], [21]. No other change in the inclusion or exclusion criteria was deemed necessary. The SC also added ECG monitoring at the week 3 visit, when the target dose of 30 mg/day was expected to have been reached in most participants, and also at the first visit after a dose increase to 30 mg for participants on slower titrations. Other ECGs could also be performed at any time at the discretion of the study physician. Electrolytes were already being regularly monitored in CitAD, but magnesium was added to the set of measures. These modifications were reviewed and approved by the DSMB, funding agency and by the ethics committee at each center and became effective in November 2011, after 142 CitAD participants had already been enrolled.

Following the second revision to the citalopram label in March 2012, the SC reviewed the new label and decided to make no further changes to the study entry criteria and procedures. However, the template consent form was revised to state that the dose of citalopram used in CitAD was higher than the FDA maximum recommended dose for people over 60.

### Statistical analyses

The CitAD sample size requirements were based on the primary outcomes and have been published [19], [20]. Enrollments following the introduction of the new ECG monitoring procedures in 2011 continued to be balanced between the citalopram and placebo groups. All participants were counted in the group to which they were assigned regardless of treatment adherence (“intention-to-treat”). Baseline characteristics of participants enrolled during the entire trial and for those enrolled after ECG monitoring began are summarized by treatment group using means and standard deviations for continuous variables and counts and proportions for categorical variables.

For participants enrolled after November 2011, QTc at week 3 was compared by treatment group using linear regression controlling for baseline QTc and 95% confidence intervals and t-tests were calculated. A similar model for change from baseline in QTc was created and a treatment by enrollment QTc interaction was included to test for differences in relationship between enrollment QTc and change in QTc by treatment group. We defined “prolonged” QTc as QTc>450 ms for men or 470 ms for women [4], [21]. “Clinically significant increase” in the QTc was defined as an increase ≥30 ms from enrollment to week 3, consistent with FDA guidance [22]. The proportion of patients with prolonged QT and change in QTc≥30 ms from enrollment to week 3 were compared by Fisher's exact test. These analyses were not planned at the beginning of the trial because the FDA advisory and corresponding protocol changes occurred well into the second half of the trial.

Statistical analyses were performed using SAS version 9.2; copyright © 2002–2008 by SAS Institute Inc Cary, NC, USA, and R version 2.13.1; copyright © 2011 by the R Foundation for Statistical Computing. All p-values are two-sided and p<0.05 was used as the threshold for statistical significance.

## Results

**Figure 1:**
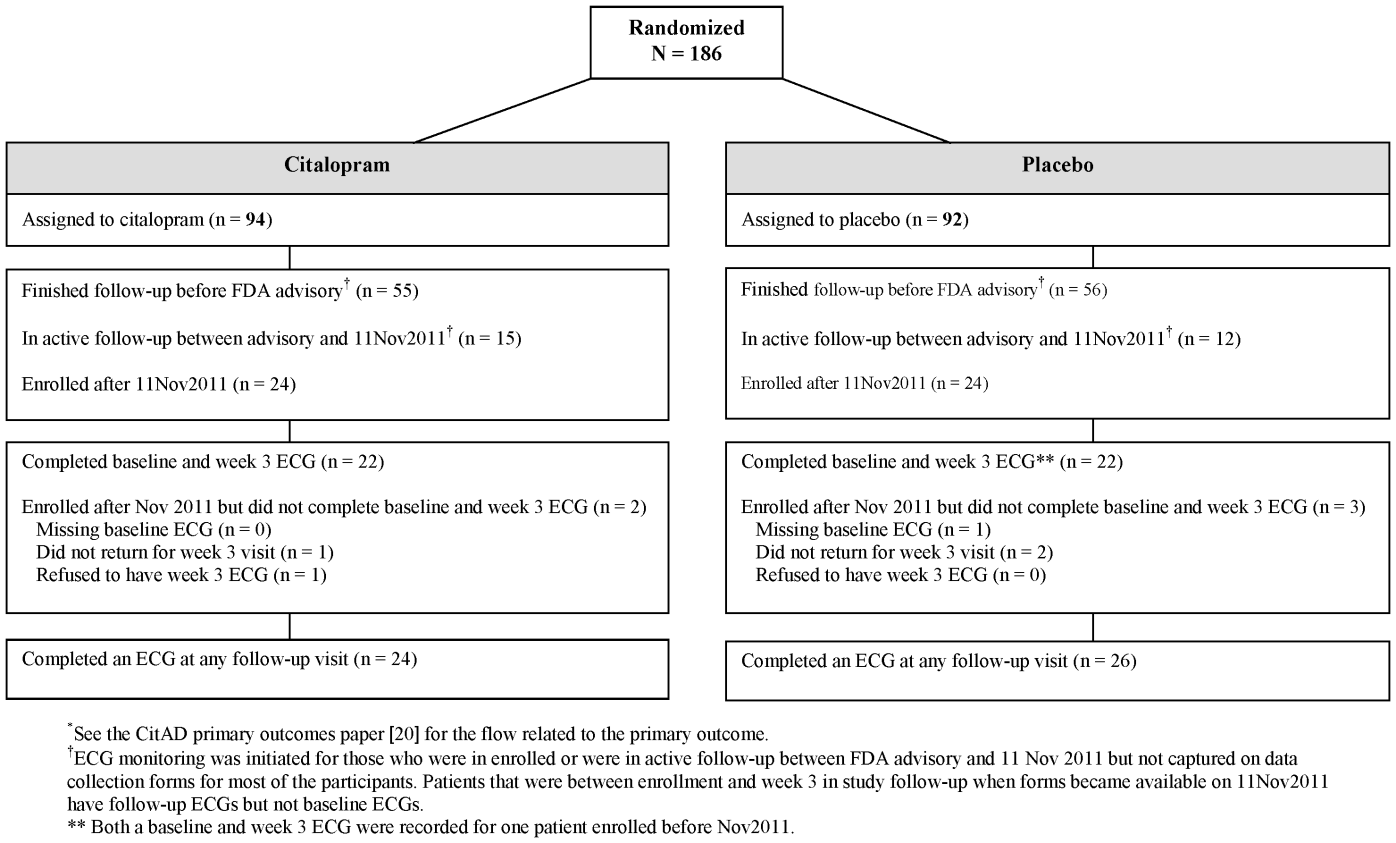
Participant flow* related to ECG monitoring.

CitAD stopped enrolling participants shortly after the planned stopping date and enrolled 186 participants in total (94 in citalopram and 92 in placebo), 48 (24 in citalopram and 24 in placebo) of which were enrolled after the ECG monitoring began. The original target enrollment was 200 but was not met due to slow enrollment at the end of the trial. The flow of participants with respect to ECG monitoring is shown in Figure 1. Between the August 2011 FDA advisory and November 2011, 27 patients who were in active follow-up were monitored informally while protocol changes were drafted and reviewed by the DSMBs and ethics committees and subsequently implemented. We did not require that ECGs performed during this interim period be retrospectively recorded on data collection forms, although some were captured. In November 2011 changes were formally implemented, the patients currently in active follow-up (n = 3) and the 48 subsequently enrolled patients had ECG recorded as described previously. However, one participant was enrolled without an ECG, one patient refused the week 3 ECG and three patients missed the week 3 visit.

Baseline characteristics of all participants as well as the subset that enrolled after new ECG monitoring procedures were implemented are shown in Table 1. The mean age of all participants at enrollment was 78 (SD = 8). Slightly less than half (85; 46%) of the participants were women. For more information on baseline characteristics see Porsteinsson, et al [20]. More than half (n = 111, 60%) of the participants had a history of hypertension at enrollment and 15 (8%) reported a previous myocardial infarction. The mean systolic and diastolic blood pressures were 132 (SD = 17) and 73 (SD = 11), respectively, with a mean resting pulse of 68 (SD = 10). There were no differences in the cardiovascular related baseline characteristics between the treatment groups. The mean baseline QTc interval was 417 milliseconds (ms) (SD = 22) and was not different between the two treatment groups.

**Table 1:** Baseline characteristics of the patients.

	*All participants*						*Enrolled after ECG monitoring began* *					
	Total		Citalopram		Placebo		Total		Citalopram		Placebo	
No. randomized	186		94		92		48		24		24	
Age in years, mean (SD)	78	(8)	78	(9)	79	(8)	75	(9)	74	(10)	76	(9)
Women, n (%)	85	(46%)	44	(47%)	41	(45%)	21	(44%)	10	(42%)	11	(46%)
Racial/ethnic group, n (%)												
White, non-Hispanic	120	(65%)	62	(66%)	58	(63%)	35	(73%)	18	(75%)	17	(71%)
African-American, non-Hispanic	31	(17%)	15	(16%)	16	(17%)	5	(10%)	1	(4%)	4	(17%)
Hispanic/Latino	24	(13%)	10	(11%)	14	(15%)	5	(10%)	3	(13%)	2	(8%)
Other, non-Hispanic	11	(6%)	7	(7%)	4	(4%)	3	(6%)	2	(8%)	1	(4%)
Highest education, n (%)												
No High school diploma	52	(28%)	25	(27%)	27	(29%)	9	(19%)	5	(21%)	4	(17%)
High school diploma	43	(23%)	20	(21%)	23	(25%)	13	(27%)	4	(17%)	9	(38%)
Some college/associates degree	29	(16%)	18	(19%)	11	(12%)	7	(15%)	5	(21%)	2	(8%)
Bachelor's degree	37	(20%)	21	(22%)	16	(17%)	14	(29%)	9	(38%)	5	(21%)
Professional/graduate degree	25	(13%)	10	(11%)	15	(16%)	5	(10%)	1	(4%)	4	(17%)
Concomitant medications, n (%)												
Aspirin	80	(43%)	43	*(46%)	37	(40%)	20	(42%)	10	(42%)	10	(42%)
Statins	80	(43%)	43	(46%)	37	(40%)	20	(42%)	11	(46%)	9	(38%)
Thiazide diuretics	29	(16%)	11	(12%)	18	(20%)	6	(13%)	1	(4%)	5	(21%)
ACE inhibitors	47	(25%)	25	(27%)	22	(24%)	12	(25%)	6	(25%)	6	(25%)
Beta-blockers	36	(19%)	21	(22%)	15	(16%)	11	(23%)	7	(29%)	4	(17%)
QT interval, mean (SD)		na		na		na	417	(22)	419	(17)	416	(27)
History** of hypertension, n (%)	111	(60%)	61	(65%)	50	(54%)	26	(54%)	12	(50%)	14	(58%)
History** of myocardial infarction, n (%)	15	(8%)	9	(10%)	6	(7%)	4	(9%)	3	(14%)	1	(4%)
Blood pressure												
Systolic, mean (SD)	132	(17)	135	(17)	129	(17)	132	(16)	136	(14)	128	(16)
Diastolic, mean (SD)	73	(11)	74	(10)	71	(11)	73	(11)	75	(11)	71	(11)
Resting pulse, mean (SD)	68	(10)	67	(11)	69	(9)	67	(11)	66	(11)	69	(10)

*ECG monitoring began on 11Nov2011 following the FDA safety communication regarding citalopram and QT prolongation.

**Self or caregiver reported history.

**Figure 2:**
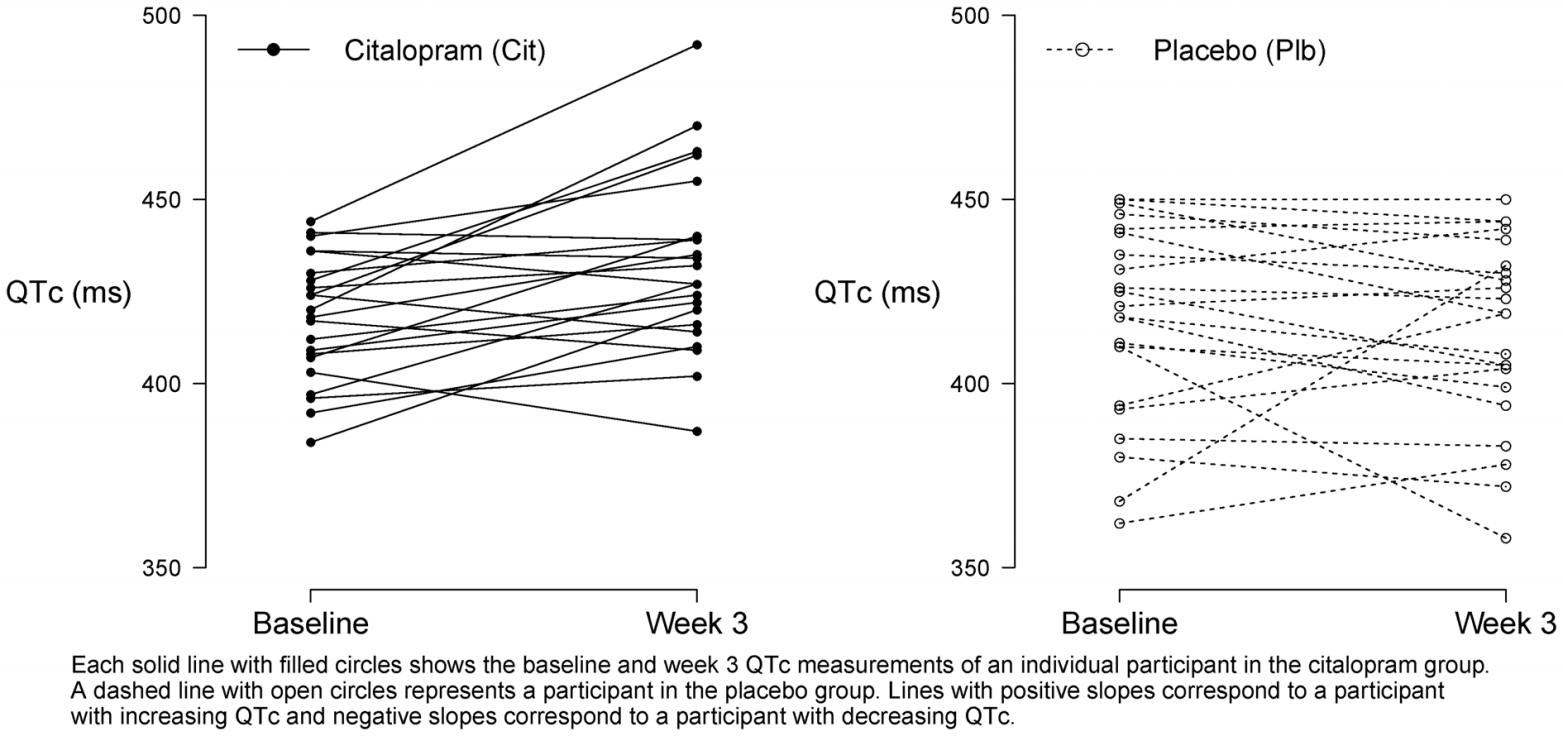
Baseline and week 3 QTc.

Forty-four participants had ECGs at both baseline and week 3. Of these, 31 participants (16 in citalopram, 15 in placebo) were on 3 study capsules per day at the week 3 ECG, 7 (3 in citalopram, 4 in placebo) were on 2 capsules, 4 (2 in citalopram, 2 in placebo) were on 1 capsule, 1 (in citalopram) was on 0 capsules and 1 (in placebo) was missing dose information at week 3. The raw QTc data are shown in Figure 2 and the analysis results are shown in Table 2. The mean QTc at week 3 was 432 ms (SD = 24) in the citalopram group and 415 ms (SD = 25) in the placebo group. The mean change in QTc from enrollment to week 3 (for all participants having both measurements regardless of dose) was 14.9 ms (SD = 19) in citalopram and −2.9 ms (SD = 22) in placebo. In regression analysis, citalopram was associated with a longer QTc interval at week 3 compared to placebo; the difference in week 3 QTc for citalopram compared to placebo adjusting for enrollment QTc was 18.1 ms (95% CI: 6.1, 30.1; p = 0.004) [20]. The results were unchanged after excluding the one citalopram participant enrolled after ECG monitoring began that was no longer taking citalopram at the week 3 ECG.

**Table 2:** ECG monitoring.

	Total		Citalopram		Placebo		p-value
Number of participants enrolled after ECG monitoring began	48		24		24		
Number of participants with at least one follow-up ECG	50		24		26		
*At least one prolonged QT interval during follow-up* *, *n (%)*	*4*	*(8%)*	*3*	*(13%)*	*1*	*(4%)*	*0.340*
Number of participants with enrollment and week 3 ECG	44		22		22		
Mean (SD) QT interval at week 3	424	(26)	432	(24)	415	(25)	
Mean (SD) change in QT interval from enrollment to week 3	6.0	(22)	14.9	(19)	−2.9	(22)	
*Difference in QT interval (Cit - Plb) at week 3* **	*18.1*	*(95% CI: 6.1, 30.1)*					*0.004*
*Increase in QT interval* ≥*30 ms* *, *n (%)*	*8*	*(18%)*	*7*	*(32%)*	*1*	*(5%)*	*0.046*

*P-value calculated using Fisher's exact test.

**P-value calculated using linear regression of week 3 QT interval on treatment group controlling for baseline QT interval. Positive difference indicates longer QTc interval in citalopram.

Almost one third of the participants in the citalopram group (n = 7, 32%) had a clinically significant increase of greater than 30 ms from enrollment to week 3 compared to only 1 (5%) in the placebo group (Fisher's exact p = 0.046) [20]. Of the patients with any follow-up ECGs (n = 50), only four patients met the gender-specific threshold for prolonged QTc at any point during follow-up (3 [13%] in citalopram versus 1 [4%] in placebo, Fisher's exact p = 0.611) [20]. Although the number of participants on a dose lower than 3 caps per day was small, it is notable that 2 of the 3 citalopram participants on 20 mg/day and 1 of the 2 citalopram participants on 10 mg/day had an increase ≥30 ms from baseline to 3 weeks.

**Figure 3:**
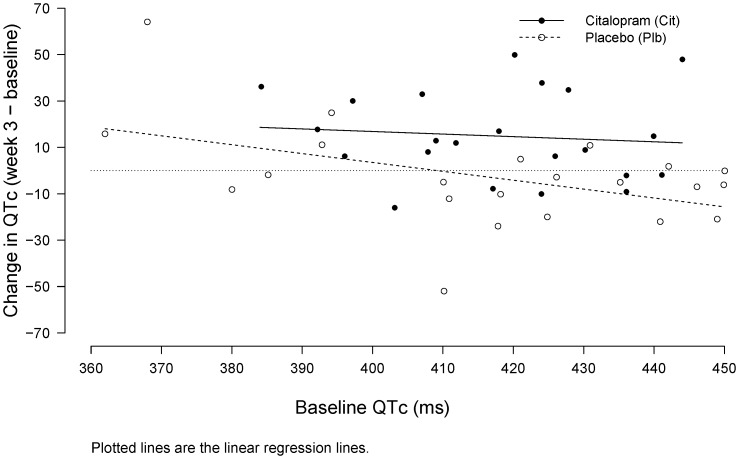
Change in QTc by baseline QTc and treatment group.

Changes in QTc by baseline QTc and treatment assignment are shown in Figure 3. The change in QTc was inversely related to the enrollment QTc (combined [citalopram and placebo] slope estimate = −0.293 [95% CI: −0.593, −0.007], p = 0.055) suggesting higher enrollment QTc was associated with lower change in QTc, possibly due to regression to the mean but also influenced by the placebo outlier (see Figure 3, top right corner of figure). However, the relationship between enrollment QTc and change in QTc did not differ statistically between treatment groups (interaction p = 0.372), indicating that the difference in change in QTc between treatment groups was fairly constant across varying values of enrollment QTc. Results with the aforementioned influential point removed are available in the Text S1 and Figure S1.

**Figure 4:**
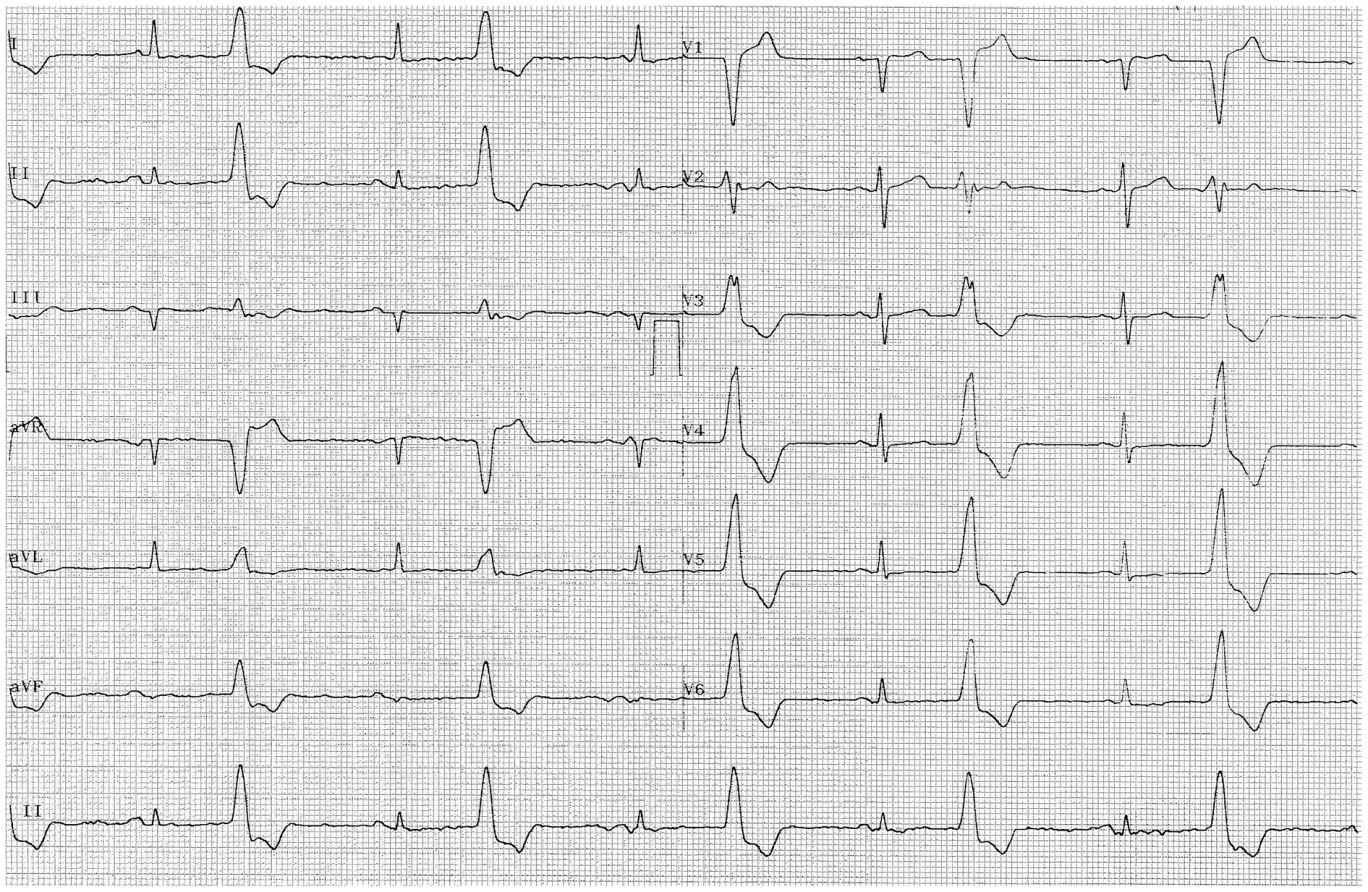
ECG of patient with prolonged QTc and bigeminy.

One of the men in the citalopram group that displayed prolonged QTc at week 3 (492 ms) also showed bigeminy on the ECG. The ECG is shown in Figure 4. This participant had a baseline QTc of 444 ms and was also taking alfuzosin which is known to lengthen QT (QTdrugs.org). The study cardiologist (masked to treatment group) recommended stopping study drug after the week 3 ECG and the patient continued follow-up off of drug without any complications but no follow-up ECGs are available.

Two participants in the citalopram group had serious adverse events labeled as ‘syncope’ that required hospitalization. The syncope events occurred before ECG monitoring began so no QTc data are available. No syncope events were reported in the placebo group; however, one patient in the placebo group fell and broke her hip while reaching for belongings on a dresser. More information about serious adverse events is available in the primary report of CitAD [20]. No participants in either group had a cardiovascular related death or other cardiovascular related serious adverse events. The single reported death, in the placebo group, was from a previously undetected lung cancer.

## Discussion

The mean QTc for CitAD participants at enrollment was similar to that seen in other studies of older adults [23]–[26]. Compared with placebo, the increase in QTc interval for citalopram was approximately 18 ms longer and the difference between the groups was independent of the baseline QTc value. This increase is higher than what we would expect based on the data in the FDA drug safety communication [10] but older patients have diminished clearance of citalopram; in a pharmacokinetic analysis of 106 subjects (ages 22–93), investigators found that citalopram clearance decreased 0.23 L/h for every year of age [27].

Although the study was not designed to examine differences in QTc, the blocked randomization scheme produced comparable groups after the ECG monitoring procedures were added. In the placebo group, the difference between the baseline and week 3 QTc intervals were centered close to zero (at −3 ms) indicating fairly stable measurements on average, although one participant had a large change in QTc in the placebo group. Any noise added because of inherent variability in ECG reads or in differing QT to QTc conversions, or because the ECGs were done at different times of the day [4], [5] should be equally distributed in the citalopram and placebo groups, and in fact, variance estimates were similar in the two groups. The lack of standardization of QTc calculation could affect the classification of ‘prolonged QT’ although the mean resting heart rate in the CitAD participants was 68 (SD = 10) and the various formulae give similar conversions in this range [3]. This lack of precision in estimating QTc also reflects the reality of using this equipment and is important for generalization to clinical practice.

QT prolongation is a surrogate measure for arrhythmia [1]. Many drugs are known to cause QT prolongation and the degree of QT prolongation caused by citalopram in the current study was comparable with increases in QTc due to other drugs known to interfere with myocardial repolarization [28], [29]. FDA guidance for industry states that drugs that are associated with mean increases of >20 ms “have a substantially increased likelihood of being proarrhythmic” [22] and our observed difference is approaching that magnitude of increase. Whether citalopram was directly responsible for any adverse clinical outcomes in our cohort is unclear. A study with a larger sample size would be needed to determine if these changes in QTc were indicative of changes in incidence of arrhythmia and cardiac events. Even in this small sample, two patients in the citalopram group had unexplained syncope (prior to the onset of ECG monitoring) and one citalopram patient experienced profound QTc prolongation and bigeminy (frequently a harbinger of impending TdP), suggesting the possibility of a real and worrisome citalopram-induced effect. The latter patient was also taking alfuzosin, a drug that should be used with caution in patients who are taking other medications that prolong the QT interval, such as citalopram [30]. We did not allow enrollment of men with QTc>450 or women with QTc>470 after the FDA advisory so it is possible that we are underestimating the proportion of older adults who might develop prolonged QTc after initiating citalopram.

Our results provide data to support the FDA warning against the use of citalopram in doses higher than 20 mg/day in people over 60. Citalopram, at 30 mg/day, was associated with improvement in agitation in patients with AD but also with concerning cognitive effects and QT prolongation.

Resource center representatives:

## Supporting Information

Figure S1 **Change in QTc by baseline QTc and treatment group excluding influential point.**
(TIF)

Protocol S1 **CitAD protocol.**
(PDF)

Checklist S1 **CONSORT checklist.**
(DOC)

Text S1 **Sensitivity analyses supporting information.**
(DOCX)
