# Supplementary material for: Changes in QTc Interval in the Citalopram for Agitation in Alzheimer's Disease (CitAD) Randomized Trial
Source: PLoS One. 2014 Jun 10;9(6):e98426. doi: 10.1371/journal.pone.0098426 (PMC4051660; doi:10.1371/journal.pone.0098426)
Supplement: Protocol S1 — CitAD protocol. (PDF) [file pone.0098426.s002.pdf]

Citalopram for Agitation in Alzheimer's Disease (CitAD)

CitAD Protocol

Version 2.3

Repository:
CitAD Coordinating Center
Johns Hopkins University
911 South Ann Street
Baltimore, Maryland 21231
(443) 287-3170
(443) 287-5797 (fax)

Citalopram for Agitation in Alzheimer's Disease (CitAD)

CitAD Protocol

Version 2.3
Version date: 5 October 2011
Distribution date: 12 October 2011

Repository:
CitAD Coordinating Center
Johns Hopkins University
911 South Ann Street
Baltimore, Maryland 21231
(443) 287-3170
(443) 287-5797 (fax)

Document distribution

Version	Version date	Distribution	Distribution Date
0.0 (draft)	4 Sep 08	Steering Committee at investigators' meeting	10 Sep 08
1.0 (official)	15 Oct 08	Steering Committee, Lead Coordinators, and Coordinating Center, via PPM	16 Oct 08
1.1 (draft)	6 May 09	Steering Committee and Coordinating Center, via e-mail	6 May 09
1.2 (draft)	28 May 09	Steering Committee and Coordinating Center, via e-mail	28 May 09
2.0 (official; withdrawn)	02 Jun 09	Steering Committee, Lead Coordinators, and Coordinating Center, via PPM	02 Jun 09
2.0 (official)	19 Jun 09	Steering Committee, Lead Coordinators, and Coordinating Center, via PPM	22 Jun 09
2.1 (official)	18 Oct 10	Steering Committee, Lead Coordinators, and Coordinating Center, via PPM	18 Oct 10
2.2 (official)	10 Dec 10	Steering Committee, Lead Coordinators and Coordinating Center, via PPM	10 Dec 10
2.3 (draft)	22 Sep 11	Steering Committee and Coordinating Center, via e-mail	23 Sep 11
2.3 (official)	5 Oct 11	Steering Committee and Coordinating Center, via PPM	12 Oct 11

CitAD protocol

Document history

Version 0.0 Draft (4 Sep 08)

Version 1.0 Official (15 Oct 08)

Changes from protocol version 0.0 to 1.1
Study procedures relating to drug close, titration and termination, assessments used during telephone contacts and lorazepam use were updated

Version 1.1 Draft (6 May 09)

Changes from protocol version 1.0 to 1.1:
Minor editorial and wording changes were made throughout the document to improve clarity. Substantive modifications were made to the following sections:

Summary:
- List of the effects of citalopram that will be examined was modified to exclude quality of life

2 Objectives:
- Side effects related to citalopram will be monitored as opposed to all psychotropic effects

4 Eligibility criteria:
- Minor editorial and wording changes were made to improve clarity.
- Inclusion criteria "Sufficient fluency, of the patient and the caregiver, in written and spoken English or Spanish" was modified to "Sufficient fluency, of the patient and the caregiver, in written and spoken English"

7.3 Enrollment Visit:
- Following patient assessments were deleted to reduce the patient burden at the time of enrollment visit:
  - Cognitive subscale of the Alzheimer's disease Assessment Scale (ADAS-Cog)
  - Alzheimer's Disease Related Quality of Life (ADRQL)
  - Udvalg for Klinske Undersolger (UKU)

8.2 Scheduled in-person visits:
- Following patient assessments were deleted to reduce the patient burden at the time of follow-up visit:
  - Cornell Scale of Depression in Dementia (CSDD)
  - Alzheimer's Disease Related Quality of Life (ADRQL)
  - Udvalg for Klinske Undersolger (UKU)

- Cognitive subscale of the Alzheimer's disease Assessment Scale (ADAS-Cog), at week 9 only

8.5 Assessments by visit table updated to reflect changes in assessments

9 Outcome assessment and neuropsychological testing:

- Details on the following assessments, which will not be administered to participants under revised protocol were deleted :
  - Alzheimer's Disease Related Quality of Life (ADRQL)
  - Cognitive subscale of the Alzheimer's disease Assessment Scale (ADAS-Cog)
  - Udvalg for Klinske Undersolger (UKU)

10.3 Analysis of secondary outcomes:

- Patient outcomes on Alzheimer 's disease related Quality of Life (ADRQL), Cornell Scale of Depression in Dementia (CSDD), cognitive domains rated by ADAD-Cog will not be assessed
- Side effects rating will be monitored by medical monitoring of adverse events indicated for citalopram as opposed to Udvalg for Klinske Undersolger (UKU) scale

13.4 Consent procedures for DNA blood collection:

- Consent procedures for DNA collection, electrolyte panels, and citalopram levels were modified to "Consent procedures for DNA blood collection" to clarify that consent procedures for DNA blood collection procedures are optional

15 Literature cited:

- Following citations were deleted:
  - Citation number 18 (Gonzales-Salvador T, Lyketsos CG, Baker AS, Roques C, Hovanek L, Steele CD, Brandt J. Quality of life of patients with dementia in long-term care. Int J Ger Psychiatry 2000; 15: 181-189)
  - Citation number 25 (Lingjaerde O, Ahlfors UG, Bech P, et al, The UKU Side Effect Rating Scale: a new comprehensive rating scale for psychotropic drugs and cross-sectional study of side effects in neuroleptic treated patients. Acta Psychiatr Scand Suppl 1987; 334: 1-100)
  - Citation number 30 (Lyketsos CG, Gonzales-Salvador T, Chin JJ, Baker A, Black B, Rabins PV. A follow-up study of change in quality of life among persons with dementia residing in a long-term care facility. Int J Ger Psychiatry 2003; 18:275-281)
- Citation references were rearranged throughout the document

Appendix 1:

- Title "Appendix 1" was changed to "Appendix-1: Power calculations" for clarification
- Sample size calculation description and corresponding table regarding Udvalg for Klinske Undersolger (UKU) scale were deleted

Appendix 2: CitAD design summary:

- Primary outcome measures, other outcome measures, and related hypothesis were added to design summary.
- Word "psychotropic" was deleted from the secondary objectives
- Inclusion criteria "Sufficient fluency, of the patient and the caregiver, in written and spoken English or Spanish" was modified to "Sufficient fluency, of the patient and the caregiver, in written and spoken English"
- Outcome "Difference of cognitive, function, and quality of life measures" was modified to "Difference in cognitive and physical function measures"

Version 1.2 Draft (28 May 09)

Changes from protocol version 1.1 to 1.2

4 Eligibility criteria:

- Inclusion criteria related to clinically significant agitation was modified to clarify meaning:
    - "a medication is needed in the opinion of study physician" was modified to "a medication for agitation is appropriate in the opinion of study physician"
    - "score >= 4 on the agitation domain, score > 2 agitated behaviors per week as assessed on the NPI" were modified to “either the frequency of agitation as assessed by the NPI is ‘Very frequently’ or the frequency of agitation as assessed by the NPI is ‘Frequently’ and severity of the agitation as assessed by the NPI is ‘Moderate’ or ‘Marked’ ”

7.3 Enrollment Visit:

- Patient assessment "Modified AD Cooperative study -Clinical Global Impression of Change" was modified to "Modified AD Cooperative study -Clinical Global Impression Worksheet"

Appendix 2: CitAD design summary:

- Inclusion criteria related to clinically significant agitation was modified to improve the clarity
    - "a medication is needed in the opinion of study physician" was modified to "a medication for agitation is appropriate in the opinion of study physician"
    - "score >= 4 on the agitation domain, score > 2 agitated behaviors per week as assessed on the NPI" were modified to "the frequency of agitation as assessed by the NPI is ‘Very frequently’ or the frequency of agitation as assessed by the NPI is ‘Frequently’ AND the severity of the agitation as assessed by the NPI is ‘Moderate’ or ‘Marked’ ”

Version 2.0 Official (19 Jun 09)

Changes from protocol version 1.2 to 2.0

4 Eligibility criteria:

- Minor wording and editorial changes were made to improve clarity in inclusion criteria. “Provision of informed consent for participating in the study by patient, caregiver, or surrogate (if necessary)” was modified to “Provision of informed consent for participation in the study by patient or surrogate (if necessary) and caregiver”

- Inclusion criteria of “Sufficient fluency, of the patient and caregiver, in written and spoken English to participate in study visits, neuropsychological testing, and other outcome assessments” was removed

9 Outcome assessment and neuropsychological testing

- Neurobehavioral Rating Scale (NBRS) will be rated by both caregiver and patient

13.3 Consent procedures

- Description of consent procedures were modified to emphasize that caregiver consent is necessary

Appendix 2: CitAD design summary:

- Minor wording and editorial changes were made to improve clarity in inclusion criteria. “Provision of informed consent for participating in the study by patient, caregiver, or surrogate (if necessary)” was modified to “Provision of informed consent for participation in the study by patient or surrogate (if necessary) and caregiver

- Inclusion criteria of “Sufficient fluency, of the patient and caregiver, in written and spoken English to participate in study visits, neuropsychological testing, and other outcome assessments” was removed

Version 2.1 Official (18 Oct 10)

Changes from protocol version 2.0 to 2.1

2. Objectives

- “To examine the effects of citalopram treatment for agitation, without depression, on other critical outcomes”, was modified to “To examine the effects of citalopram treatment on function and cognition of the patient as well as caregiver burden”
- Added “major” in primary objective

4 Eligibility criteria

- Inclusion criterion “If on treatment for AD, stability of treatment (i.e., memantine or cholinesterase inhibitors), no changes to AD medications (e.g., starting or stopping treatment) in the 3 months prior to randomization, with the exception that dose adjustments within the therapeutic range (i.e., total daily dose of ≥10 mg of memantine, ≥5 mg of donepezil, ≥16 mg per day of galantamine, or ≥4 mg per day of rivastigmine) are allowed as long as

1) the patient has received the minimum therapeutic range dose or higher for at least 3 months;
2) the last dose change was at least 1 month prior to randomization; and
3) no further dose adjustments are planned during the study's treatment phase”

was changed to “No change to AD medications within the month preceding randomization, including starting, stopping, or dosage modifications”

- Exclusion criterion “Current treatment with antipsychotics, anticonvulsants, other antidepressants (other than trazodone, ≤50 mg per day at bedtime), benzodiazepines (other than lorazepam), or psychostimulants” was modified to “Current treatment with antipsychotics, anticonvulsants (other than dilantin), other antidepressants (other than trazodone ≤50 mg per day at bedtime), benzodiazepines (other than lorazepam), or psychostimulants”

5 Randomization and masking
- Deleted “contact the CC to”
- Added “through the data system”
- Replaced “CC” with “data system”

6.3 Psychosocial treatment
- Deleted “A copy of the JHU- Dementia Care guidelines for caregivers”

6.4 Adherence to study treatment
- “Patients and /or caregivers will be asked to return all study medicines and bottles used and unused at each visit” was modified to “Patient and /or caregivers will be asked to return all study bottles with any unused capsules at each visit”
- Added “Bottles are to be returned even if all study drug was used”

6.5 Management and reporting of adverse events
- Replaced “these by body system (e.g., gastrointestinal, nervous system, etc.) with “adverse events”
- Replaced “contacts” with “visits”
- Deleted “and to the manufacturer of citalopram”

6.6 Concomitant medications
- Added “(other than dilantin)”
- Revised “(i)” to be consistent with eligibility criteria

8.6 Data collection by visit
- Section header added to version 2.1

10.2 Analysis of primary outcomes

- The mADCS-CGIC seven point scale was changed to "(1 = marked improvement, 2 = moderate improvement, 3 = minimal improvement, 4 = no change, 5 = minimal worsening, 6 = moderate worsening, 7 = marked worsening)"

10.3 Analysis of secondary outcomes

- Removed "in the first 2 weeks" from "Total dose of lorazepam used in the first 2 weeks will also be recorded"

12.3 Training of staff

- Replaced "manual" with "materials"

13.2 Monitoring of IRB approvals

- Deleted "or Chairman's office"

Appendix 1: Power calculations

- Changed "three hypotheses" to "two hypotheses"
- Deleted "and citalopram is clinically equivalent to placebo on the UKU scale"
- Deleted "and third" from "For the first and third hypotheses..."

Appendix 2: CitAD design summary

- Updated primary and secondary objectives per the changes made in section 2
- Revised "Other outcomes" section for clarity
- "Study population" section was modified to "200 patients who meet the CitAD criteria for clinically significant agitation"
- Updated inclusion and exclusion criteria to be consistent with section 4

Version 2.2 Official (10 Dec 10)

Changes from protocol version 2.1 to 2.2

4 Eligibility criteria

- Inclusion criterion related to MMSE was modified:
  - "MMSE score of 5 - 26 inclusive" was changed to "MMSE score of 5-28 inclusive"

9 Outcomes assessment and neuropsychological testing

- The 7-point Likert scale of the mADCS-CGIC was modified to reflect previous changes
  - "much better" was changed to "marked improvement"
  - "much worse" was changed to " marked worsening"

15 Literature cited

- Added citation number 25 (Livingston G, Johnson K, Katona C et al. Systematic review of psychological approches to the management of neuropsychiatric symptoms of dementia. Am J Psychiatry 2005; 162: 1996-2021)

- Citation references were rearranged throughout the document

Version 2.3 Draft (22 Sep 11)

Changes from protocol version 2.2 to 2.3:

Minor editorial and wording changes were made throughout the document to improve clarity. Substantive modifications were made to the following sections:

1.6 Safety of SSRIs

- Added "In August 2011 the FDA released an announcement[15] indicating that citalopram causes dose-dependent QT interval prolongation"

4 Eligibility criteria

- Added exclusion criterion "Prolonged QT interval"

6.5 Management and reporting of adverse events

- Added "ECGs" to the list of safety assessments

7.3 Enrollment visit

- Added "Perform an electrocardiogram (ECG)" to the list of baseline assessments

8.2 Scheduled in-person visits

- Added "Perform an electrocardiogram (ECG) (at week 3 for all patients and an additional ECG at next scheduled visit for patients whose dose is increased to 3 capsules after week 3)
- Added "Discuss adverse events"
- Revised "Review compliance and adverse events" to "Review visit schedule and compliance"
- Revised "Refer the patient to a study physician if the patient exhibits a notable change in condition or is medically unstable" to add "primary care physician, or specialist"

8.4 Telephone contacts

- Added "Review visit schedule"
- Added "Collect adverse events"

8.6 Data collection by visit

- Added "Perform electrocardiogram (ECG)" to list of procedures

9 Outcomes assessment and neuropsychological testing

- Added "magnesium levels" to the electrolyte panel

13.5 Potential risks and benefits

- Added "In August 2011 the FDA announced that citalopram was associated with a dose dependent QT prolongation and should not be used at doses over 40 mg per day.[15] The data

the FDA used to support the announcement did not include any patients over 50 years of age (Thomas Laughren (FDA), personal communication). The increase in the QT internal at 40 mg was estimated to be 12.6 ms with 90% confidence intervals ranging from 10.9 to 14.3 ms.

15 Literature cited

- Added citation number 15 (Food and Drug Administration website (2011): http://www.fda.gov/Drugs/DrugSafety/ucm269086.htm)

Version 2.3 Official (5 Oct 11)

Contents

Document distribution. i

Document history. ii

Summary. xii

1. Background and significance. 1
    1.1. Public health significance of Alzheimer's disease. 1
    1.2. Neuropsychiatric symptoms of Alzheimer's disease. 1
    1.3. Agitation in Alzheimer's disease. 1
    1.4. Treatment options for agitation in AD are limited. 2
    1.5. Rationale for using SSRIs 2
    1.6. Safety of SSRIs. 2
    1.7. Rationale for CitAD. 3
    1.8. Rationale for selection of citalopram. 4

2. Objectives. 5

3. Design. 6
    3.1. Design features. 6
    3.2. Sample size and power. 6

4. Eligibility criteria. 7

5. Randomization and masking. 9

6. Treatment plan. 11
    6.1. Treatment groups. 11
    6.2. Treatment schedule and titration of study drug dose. 11
    6.3. Psychosocial treatment. 11
    6.4. Adherence to study treatment. 12
    6.5. Management and reporting of adverse events. 12
    6.6. Concomitant medications. 13
    6.7. Assessment of suicidality and need for hospitalization. 13

7. Enrollment visit. 14
    7.1. Overview of recruitment. 14
    7.2. Overview of consent issues. 14
    7.3. Enrollment visit. 14

8. Follow-up visits and telephone contacts. . . . 16
   8.1. Overview of follow-up visits. . . . 16
   8.2. Scheduled in-person visits. . . . 16
   8.3. Citalopram levels and electrolyte panels. . . . 16
   8.4. Telephone contacts. . . . 17
   8.5. Unscheduled follow-up visits and contacts. . . . 17
   8.6. Data collection by visit. . . . 18

9. Outcomes assessment and neuropsychological testing. . . . 19

10. Analysis plan. . . . 21
   10.1. General principles. . . . 21
   10.2. Analysis of primary outcomes. . . . 21
   10.3. Analysis of secondary outcomes. . . . 23

11. Data monitoring. . . . 25

12. Quality assurance and performance monitoring. . . . 26
   12.1. Overview. . . . 26
   12.2. Certification of sites. . . . 26
   12.3. Training of staff. . . . 26
   12.4. Certification of staff. . . . 26
   12.5. Performance monitoring. . . . 27
   12.6. Site visiting. . . . 27
   12.7. Error detection. . . . 27

13. Protection of human subjects. . . . 28
   13.1. Human subjects. . . . 28
   13.2. Monitoring of IRB approvals. . . . 28
   13.3. Consent procedures. . . . 28
   13.4. Consent procedures for DNA blood collection. . . . 29
   13.5. Potential risks and benefits. . . . 29
   13.6. Safety monitoring. . . . 31
   13.7. Confidentiality of patient and caregiver data. . . . 31

14. Biohazards. . . . 32

15. Literature cited. . . . 33

Appendix 1: Power calculations. . . . 39

Appendix 2: CitAD design summary. . . . 42

Summary

Citalopram for Agitation in Alzheimer's Disease (CitAD) is a placebo-controlled, masked, 9-week, 8-site randomized clinical trial, sponsored by National Institute of Aging and National Institute of Mental Health, involving 200 patients with Alzheimer's disease (AD) designed to examine the efficacy and safety of citalopram as treatment for clinically significant agitation in AD patients. It will also investigate pharmacogenomic, genetic, and clinical predictors of response to citalopram therapy. CitAD will enroll patients from real world setting such as outpatient, nursing home, and assisted living facilities and will examine the effects of citalopram on agitation, other neuropsychiatric symptoms, cognition, and daily functioning. CitAD will also conduct careful safety monitoring.

CitAD protocol

1. Background and significance

1.1. Public health significance of Alzheimer's disease

Alzheimer's disease (AD) is a growing public health problem whose global burden is expected to exceed 80 million cases by 2040[12]. There are 2.3 million living victims in the United States[8]. With an annual incidence of about 360,000 cases, and given the aging of the population, improvements in life expectancy and improvements in treatment of AD, the prevalence of this condition in the USA may quadruple in the next 50 years[8]. AD is a progressive disease with debilitating consequences for patients. Its hallmark is gradual cognitive decline[42], worsening over the course of a decade, with median survival time approximately 3.5 years[57]. AD debilitates both patients and families emotionally as well as economically[42]. Societal costs are about $100 billion per year in the US alone[13] and may triple by 2040[16].

1.2. Neuropsychiatric symptoms of Alzheimer's disease

While cognitive and functional decline is the hallmark of AD, neuropsychiatric symptoms (NPS) afflict almost all patients[53,28]. NPS are sometimes referred to as "behavioral disturbances" or "non-cognitive mental disturbances" which include agitation, delusions, hallucinations, depression, sleep disturbance, and problem behaviors. NPS add significant disability for patients and caregivers[42,29]. A range of associated adverse consequences have been reported including worse quality of life, greater disability, accelerated cognitive or functional decline, greater burden on caregivers, earlier institutionalization, and accelerated mortality[42]. Practice guidelines developed for the treatment of AD consistently refer to management of NPS as central to the treatment of AD (e.g., American Psychological Association Practice Guidelines for the Treatment of AD (revised), in press; American Association for Geriatric Psychiatry Position Statement on Principles of Dementia Care, 2006). The importance of NPS in the context of AD has also been recognized by the Food and Drug Administration (FDA), which is open to considering new indications for treating of specific NPS in this context [22]. Despite this, the empirical evidence supporting pharmacological and non-pharmacological interventions for NPS is sparse[11,29].

1.3. Agitation in Alzheimer's disease

One of the most frequent and problematic NPS associated with AD is agitation, a syndrome that involves emotional distress, excessive psychomotor activity, aggressive behaviors, irritability, and disinhibition. In a 5-year follow up of the population-based Cache County study, 42% of dementia participants developed agitation[53]. In clinical settings about 60% of AD patients develop an agitation syndrome over two years[2,46]. Agitation is a chronic and persistent problem for patients at all levels of dementia severity, particularly in the middle and later stages when the Mini Mental State Exam (MMSE) score is below 20[52,2,46].

Agitation has serious adverse effects for patients and caregivers and is a major source of disability for dementia patients[42]. In general, its effects are more harmful than those of the cognitive symptoms[29]. Agitation is a major source of impaired quality of life, caregiver burden, dangerous

behaviors, institutionalization, restraint, and psychiatric admission. Therefore the management of agitation is a major priority in caring for people with AD, as it carries a poor prognosis[29].

1.4. Treatment options for agitation in AD are limited

Given its frequent occurrence as a major clinical problem, research into the management of agitation in AD has a long history. Several pharmacologic and non-pharmacologic treatments have been assessed. Nonpharmacologic treatments have not proven effective, especially for moderate or more severe agitation[26]. Pharmacologic strategies suggest that atypical antipsychotics may have a role, but the risk benefit trade-off is substantial and therefore their utility for agitation in AD is potentially limited[47]. Other medications are similarly limited in their efficacy. Given the significant problem that agitation represents, better pharmacologic options are needed.

1.5. Rationale for using SSRIs

The neurotransmitter serotonin is an inhibitory modulator of aggression. Serotonergic dysfunction has been associated with a range of agitation-like syndromes in animal models and humans. Serotonergic loss is widespread in the brains of AD patients even in early disease stages. Serotonergic system loss, genetic variation, or dysfunction has been associated with agitation in AD patients. This evidence, coupled with early clinical trial findings suggest that addressing serotonergic loss through the use of serotonin agonists or serotonin reuptake inhibitors (SSRIs) is a reasonable approach for the treatment of agitation in AD.

1.6. Safety of SSRIs

SSRIs are commonly prescribed for AD patients but their safety has not been fully established. There are few placebo-controlled trials and the safety results are not definitive. The critical importance of safety data about medication treatments for agitation in AD, especially data allowing consideration of safety in the context of efficacy, is underscored by the recent Clinical Antipsychotic Trial Intervention Effectiveness (CATIE) experience with atypical antipsychotics[47]. Recent experience with cardiovascular and mortality risks involving use of antipsychotics in AD has raised the important question of whether AD patients, especially those with agitation, are disproportionately vulnerable to psychotropic medication risks [48,43]. Well-designed trials of SSRIs for agitation in AD are needed to allow systematic consideration of safety in light of efficacy findings.

In examining the relevant literature, there have been only two placebo-controlled trials for agitation in AD involving an SSRI. In the largest trial[14], gastrointestinal (GI) symptoms were more common on sertraline (49% versus 31% on placebo), and there was greater weight loss on sertraline. Diarrhea was 3 times more common (27% versus 12%), and anxiety (14.5% versus 7.5%) and hallucinations for any reason (13.7% versus 6.7%) twice as common with sertraline. However, there was no difference between sertraline and placebo with respect to dropouts for any reason or dropouts

due to adverse events. Pollock[41] reported better tolerability for citalopram compared to placebo in a smaller agitation trial. Trials of several SSRIs for depression in AD are only partly informative, as they deal with a different AD population, but generally small studies have reported mixed results about tolerability[37,38,39,30].

The issue of whether SSRIs worsen cognitive impairment in patients with dementia is unresolved, but a recent report from DIADS[35] suggested that sertraline does not worsen cognitive functioning. This issue is a main question for the DIADS-2 trial[32]. Other safety data regarding SSRIs are derived from randomized trials in elderly depressed patients without AD. In general patients in these trials were about 5 years younger than typical AD patients and were free of neurodegenerative disease and the rates of adverse events across different SSRIs were found to be similar for the most part. Some SSRIs have been described as having stimulating adverse events, including fluoxetine[54], paroxetine, and sertraline[49]. Hyponatremia, which may be due to inappropriate anti-diuretic hormone secretion, occurred in some patients on SSRIs[21]. In August 2011 the FDA released an announcement[15] indicating that citalopram causes dose-dependent QT interval prolongation. Postural instability and falls have been associated with SSRIs in nursing home populations[30]. CitAD is designed to survey a range of relevant clinical and laboratory data to provide detailed safety information about citalopram for agitated AD patients.

1.7. Rationale for CitAD

A conference entitled "Elderly Patients with Dementia-Related Behavioral and Psychological Symptoms: A Search for Consensus on Treatment Options, Clinical Trials Methodology and Policy" was convened by the Department of Psychiatry at Harvard Medical School (Beth Israel Deaconess Hospital) and Best Practice Project Management, Inc. on June 28-29, 2006 in Bethesda Maryland. Forty key stakeholders participated, representing leading academic centers in geriatric medicine, geriatric psychiatry, neurology, and pharmacoepidemiology; industry sponsors; U.S. Food and Drug Administration; Centers for Medicare and Medicaid Services; National Institutes of Health; medical leadership group in the nursing home industry; and advocates for patients and families with AD. The conference came to the following conclusions regarding agitation in AD (Roger E. Meyer, personal communication): 1) Agitation syndrome in AD patients, if well defined, is an appropriate target for treatment; 2) SSRIs are a leading candidate medication class for evaluation in this context in well-designed trials; 3) Safety and pharmacokinetic assessments are key to build into trials of this kind; 4) Longitudinal designs are needed with repeated measures and looks at shorter-term trajectories (e.g. weekly); 5) Studies should be powered for clinically meaningful effect sizes. The stakeholders suggested that there is a distinct patient population with AD who are predominantly agitated, overactive, aggressive, irritable, and disinhibited, who can be identified for inclusion in clinical trials, and whose level of agitation can be rated reliably on scales such as the NeuroBehavior Rating Scale (NBRS) and the Neuropsychiatric Inventory (NPI).

CATIE has suggested that when treating agitation in AD, a complex interplay exists between efficacy, safety, symptom changes, burden and quality of life. Since serotonergic dysfunction has

been associated with agitation-aggression in animal models and humans, it is surprising that there have been only two randomized, placebo-controlled trials examining the efficacy of SSRIs for agitation in AD. Neither trial aimed to target a carefully defined agitation syndrome. Rather, both selected patients with a range of NPS, and evaluated the effect of SSRIs on a range of several symptoms including agitation. In the larger trial[14], 144 patients with a score >5 on the total Neuropsychiatric Inventory (NPI), and a severity score ≥2 in at least one domain, were treated with donepezil for 8 weeks. Those patients who did not withdraw were randomized to sertraline or placebo for 12 weeks; only 24 patients were treated with sertraline. While sertraline was generally well tolerated despite more side effects, it did not appear to affect overall NPS. A secondary analysis, targeting patients with moderate or more severe agitation suggested efficacy for sertraline compared to placebo. In the smaller 17-day trial of psychiatric inpatients with more severe NPS, Pollock (2002)[41] reported that citalopram was superior to placebo for treating NPS, with the greatest efficacy for treating agitation, and that citalopram was also superior to perphenazine for treating agitation. While these two trials did not specifically target agitation syndromes, both found evidence of SSRI efficacy, especially for moderate or more severe agitation. The results provide good preliminary evidence that SSRIs are promising treatments for carefully selected patients with AD and agitation.

1.8. Rationale for selection of citalopram

Several SSRI antidepressants are marketed in the United States and were considered for this study. Given their favorable side effect profile, SSRIs are widely used in clinical practice with older people. Two of these, sertraline and citalopram, were recommended by an experts' panel as first line therapy for the treatment of depression, and as a second line treatment for agitation, in the context of AD[3]. Given our collaborative's experience with sertraline and citalopram in AD clinical trials, these were considered as strong candidates. A clinical trial reported that for the full spectrum of agitation, sertraline does not have efficacy over placebo[14]; but, there was evidence in secondary analysis that it may have efficacy for moderate or more severe agitation. Nevertheless, the preliminary data supporting the use of citalopram were felt to be stronger than the sertraline preliminary data. Another advantage of citalopram is the availability of more data from related trials on the pharmacokinetic profile in older people.

2. Objectives

Primary objective

- To examine in a masked, randomized trial the efficacy of citalopram for the treatment of clinically significant agitation, without major depression, in patients with Alzheimer's dementia

Secondary objectives

- To examine the effects of citalopram treatment on function and cognition of the patient as well as caregiver burden
- To examine the safety of citalopram for the treatment of clinically significant agitation, without depression, in patients with AD by comparing the citalopram and placebo treatment groups with respect to the following: vital signs, weight change, gait stability, side effects, electrolyte panels, and adverse event reports
- To examine predictors of response to citalopram therapy

---

3. Design

3.1. Design features

The study is a double-masked, randomized, multicenter clinical trial with two parallel treatment groups. Treatment assignment is stratified by clinical site and employs a 1:1 assignment ratio among the two treatment groups. Patients and caregivers are participants in this study. Patients, caregivers, and all site personnel, including physicians, nurses, and neuropsychologists, are masked to treatment assignment.

3.2. Sample size and power

The primary comparison for efficacy will be the intention-to-treat comparison of the longitudinal NBRS ratings. The hypothesis for this comparison is one of superiority, i.e., we expect that the citalopram group will have lower NBRS scores (less agitation) over the course of 9 weeks than the placebo group. The planned enrollment for the trial is 200 patients, 100 patients will be randomized to citalopram and 100 patients will be randomized to placebo. With 200 patients, the power to detect a 40% reduction in agitation severity (corresponding to a 3 to 5 point difference on the NBRS) is at least 85%, assuming a two-sided type I error of 5%. Detailed information about the sample size calculations can be found in Appendix 1.

---

4. Eligibility criteria

Men and women, including those in minority groups, will be included with no age restrictions. No specific laboratory screening studies will be required. "Usual care" treatment with citalopram does not require laboratory monitoring. Laboratory tests for qualifying a potential patient as having AD (e.g., brain imaging and blood and urine tests) will be obtained under usual clinical practices prior to entry which is consistent with current clinical standards and guidelines.

Inclusion criteria

- Probable Alzheimer's disease (NINCDS-ADRDA criteria), with MMSE score of 5-28 inclusive
- A medication for agitation is appropriate, in the opinion of the study physician
- Clinically significant agitation for which either
    1) the frequency of agitation as assessed by the NPI is 'Very frequently', or
    2) the frequency of agitation as assessed by the NPI is 'Frequently' AND the severity of the agitation as assessed by the NPI is 'Moderate', or 'Marked'
- Provision of informed consent for participation in the study by patient or surrogate (if necessary) and caregiver
- Availability of primary caregiver, who spends several hours a week with the patient and supervises his/her care, to accompany the patient to study visits and to participate in the study
- No change to AD medications within the month preceding randomization, including starting, stopping, or dosage modifications

Exclusion criteria

- Meets criteria for Major Depressive Episode by DSM-IV (TR) criteria
- Presence of a brain disease that might otherwise explain the presence of dementia, such as extensive brain vascular disease, Parkinson's disease, dementia with Lewy bodies, traumatic brain injury, or multiple sclerosis
- Psychosis (delusions or hallucinations) *requiring antipsychotic treatment* in the opinion of the study physician
- Prolonged QT interval
- Treatment with citalopram is contraindicated in the opinion of the study physician
- Failure of past treatment with citalopram for agitation after adequate trial at a minimally accepted dose ($\geq$20 mg/day)
- Treatment with a medication that would prohibit the safe concurrent use of citalopram, such as MAO inhibitors
- Need for psychiatric hospitalization or suicidal
- Current participation in a clinical trial or in any study that may add a significant burden or affect neuropsychological or other study outcomes

- Current treatment with antipsychotics, anticonvulsants (other than dilantin), other antidepressants (other than trazodone, $\leq$50 mg per day at bedtime), benzodiazepines (other than lorazepam), or psychostimulants
- Any condition that, in the opinion of the study physician, makes it medically inappropriate or risky for the patient to enroll in the trial

5. Randomization and masking

The Coordinating Center (CC) will generate random treatment assignment schedules using a documented program and SAS. The randomization schedule will be designed to yield an expected assignment ratio of 1:1 for citalopram and placebo. Assignments will be stratified by clinical site and schedules will employ permuted block designs, with block sizes to be determined and documented at the CC. Adjustment for residual or other imbalances in the baseline composition of the treatment groups, if needed, will be done using multiple regression techniques rather than through further stratification in the design. Treatment assignments will be masked to the patients and the personnel of the clinical sites, but not to a restricted set of personnel at the CC. The CC will also generate a list of randomly ordered medication identifiers which will be linked to the assignment schedule. Documentation of all these processes will be retained at the CC and shall be accessible only to authorized personnel.

Treatments will be assigned using an online program accessible to the clinical sites via the CitAD data system. After the entry of specified pre-randomization data, and confirmation of eligibility, each enrolled patient's ID will be irrevocably linked to the next unassigned treatment for that clinical site. The clinical site will be directed to issue a medication kit containing the proper assigned treatment from among those kits available at the site. Treatment assignments will be e-mailed in real-time to the CC. The data system will also check for and prevent duplicate assignments (same patient randomized more than once). The treatment assignment tables in the data system will be encrypted to prevent inadvertent disclosures.

Medication will be packaged according to a list of medication identifiers and corresponding content as directed by the CC to the drug packaging facility to be used for CitAD. Kits will be shipped to each site as needed in order to guarantee that upcoming treatment assignments can be supplied. This "just-in-time" system helps to maintain masking by avoiding the need for "bins" and minimizes the amount of medication requiring storage at the sites. The CC will also develop a plan for monitoring procedures within the drug packaging facility.

The procedures related to randomization of patients at the clinical sites will be as follows:

- Eligibility and baseline data will be collected on paper forms and entered into the database at the clinical sites
- The data system will confirm eligibility and then issue the next assignment as described above; each assignment will also be e-mailed to the CC
- The data system will automatically store the date and time of assignment, the identity of the clinical site staff person making the assignment, the patient's ID, and the medication identifier to be issued
- Randomization materials, including a visit schedule and allowable time windows for visits, will be generated for the clinical site

Emergency unmasking before the end of the treatment period is expected to be rare and will be accomplished via a monitored online function within the data system. Every such unmasking will be preceded by several warnings against inappropriate unmaskings, and all unmaskings will be accompanied by immediate e-mail messages to the CC. Clinical sites will be directed to contact the CC if the unmasking will or can be accomplished during working hours.

Unmasking will occur routinely for all patients at week 9, after the appropriate data collection is completed. Whenever a treatment assignment is to be unmasked, clinical personnel are to obtain the identity (citalopram or placebo) of the treatment through the data system. This information is to be provided to the patient and caregiver, and written documentation of treatment identity provided by the data system is to be placed in the patient's file.

6. Treatment plan

6.1. Treatment groups

Patients will be assigned to one of the following groups:

- Citalopram, target dose 30 mg per day (range 10-30 mg per day) given orally, and psychosocial intervention
- Placebo and psychosocial intervention

6.2. Treatment schedule and titration of study drug dose

The target study dose will be 30 mg per day provided as a single dose in the morning. Patients will start on 10 mg of citalopram daily for seven days. Then the dose will be increased to 20 mg per day for seven days, after which the dose will be increased to 30 mg per day. If necessary, study physicians will have the option of decreasing the daily dose to a minimum of 10 mg of citalopram per day after the first three weeks of study depending on tolerability. That is, some patients will have unacceptable side effects on 30 mg and will have to be reduced to a lower dose so as the continue study treatment. Reductions of dose will only be allowed for unacceptable side effects.

Unmasking for all patients will occur at the 9-week mark for the purposes of clinical care. Unmasking before the end of the 9-week period is to occur only in emergency situations (see randomization and masking section for more details).

6.3. Psychosocial treatment

All caregivers and the patients (if convenient) will be provided with a standardized psychosocial intervention modeled after the counseling strategies employed in DIADS-2.[32] The psychosocial intervention will consist of three components: a counseling session, the provision of education materials, and 24-hour availability for crises.

The counseling session, in which a trained study clinician will counsel the primary caregiver, will take place at each study visit after randomization. It will last approximately 20-30 minutes. Each counseling session will consist of the following elements:

- Review and adjustment of the patient and caregiver supportive care plans
- Emotional support and opportunity to ventilate feelings
- Counseling regarding specific caregiving skills
- Assistance with problem solving of specific issues that the caregiver brings to the sessions
- Answers for questions regarding the educational materials

The educational materials will consist of:

- A copy of the book The 36-Hour Day

The caregiver also will be provided with 24-hour phone access to the study nurse or physician for assistance with caregiving crises that may arise after hours.

6.4. Adherence to study treatment

To ensure adherence, study medication will be administered to patients under the supervision of their caregivers involved in the study. Study physicians will assess the capacity of each patient to monitor and administer treatments and will involve caregivers as needed to assure safe use of drug and proper adherence to the treatment protocol.

Adherence to assigned treatment will be monitored via participant interview at each visit and via pill counts. Patients will be asked to return all study bottles with any unused capsules at each visit. Bottles are to be returned even if all study drug was used.

6.5. Management and reporting of adverse events

Management: All adverse events occurring after randomization and during the 9-week treatment period, regardless of adherence to study treatment, will be recorded at all contacts. A list of common side effects of citalopram will be used to monitor for adverse events. At scheduled visits patients and their caregivers will be interviewed about whether the patient experienced any symptoms or side effects on the list since the last visit. Adverse events, other than what is listed as common side effects, will still be recorded. If adverse events are noted, they will be rated as mild, moderate, or severe based on their clinical severity and frequency. Finally, patients and caregivers will be asked about visits to doctors, healthcare providers, and emergency departments for other than routine care.

Site investigators will be responsible for monitoring the safety of patients. They will be responsible for appropriate medical care of patients during the study in connection with study procedures. Safety assessments will include physical exams, vital signs, ECGs, monitoring of adverse events, and monitoring and maintenance of concurrent medication records. In addition, the Study Chair or his physician designee will provide consultation to all sites regarding medical monitoring. He will carry a beeper 24-hours a day to receive protocol questions or reports of protocol violations and serious or problematic adverse events that site investigators believe should be referred to him.

Reporting: Supervising IRBs will be notified by local investigators of adverse events occurring at their institution, according to their reporting requirements. Investigators also will notify the medical monitor and CC in a timely fashion after knowledge of a death or of an event that is life-threatening, that results in hospitalization or prolongation of hospitalization, or that involves a persistent or significant disability or incapacity. Data collected regarding these serious events will include the treatment provided, outcome, and presumed relationship to study drug and will be updated as new information becomes available; a narrative description also will be provided. CC personnel will review the data and query the clinical sites for clarification, additional information, or

supporting documentation as necessary. Reports and narratives will be forwarded to all investigators for submission to IRBs as necessary. In addition, this information will be provided to the Data and Safety Monitoring Board (DSMB) as part of their safety review.

Study-wide summary statistics (not broken out by treatment group) of adverse events will be available upon request to all sites on an annual basis for submission to their local IRB.

6.6. Concomitant medications

The use of a wide variety of medications will be allowed, because this study will attempt to represent usual clinical practice. Patients will remain on medications necessary to treat medical co-morbidities. Use of Alzheimer's disease medications will be allowed: (I) if there is no change to AD medications within the month preceding randomization, including starting, stopping, or dosage modifications, (ii) if there are no current plans to change dosage or discontinue medication, and (iii) if the clinician believes that these medications are not causing or exacerbating the patient's agitation.

To make the efficacy comparison as straightforward as possible, antipsychotics (typical and atypical), anticonvulsants (other than dilantin), other antidepressants (other than trazodone, ≤50 mg per day at bedtime), benzodiazepines (other than lorazepam which is discussed below), and psychostimulants will not be allowed while patients are receiving masked study treatment. Study teams and caregivers will manage difficult clinical behaviors by psychosocial methods. There will be one exception to this rule, involving the treatment of agitation given what is known about the activity of citalopram in its development as an antidepressant (i.e., citalopram is not likely to have significant anti-agitation effects in the first 1-2 weeks of therapy). Lorazepam may be administered as necessary up to a maximum of 0.5 mg/day throughout the 9-week treatment period.

6.7. Assessment of suicidality and need for hospitalization

The assessment of suicidality or need for hospitalization will be based on psychiatric assessment at study screening. Specifically, suicidality will be assessed by patient and caregiver interview, and by mental status examination of the patient. The examiner will assess for severe hopelessness, passive death wish, suicidal statements, suicidal plan, or behavioral indicators of risk for self-harm. Need for hospitalization will be similarly assessed by a study physician. Hospitalization is typically indicated if there is imminent risk of harm due to agitation, such as refusal to eat, weight loss, violent behavior toward the caregiver, or suicidality.

7. Enrollment visit

7.1. Overview of recruitment

Patients will be typical outpatients with AD, recruited from clinical settings at the study sites. Residents of nursing homes and assisted living facilities will be candidates too. The use of diverse sites will promote representation from ethnic minority groups. The allowable range of cognitive impairment is as broad as possible, sufficient in the high end to establish dementia diagnosis, with sufficient residual on the low end to allow for the quantification of agitation symptoms.

7.2. Overview of consent issues

Issues of consent are important in this population of patients, as their capacity to give informed consent may be compromised. Consent is to be obtained from patients and their authorized legal representatives using procedures established by the individual sites and their overseeing IRBs in accordance with local law. In all cases, prospective patients with dementia will first be assessed for their ability to provide informed consent. Capacity to give consent will be assessed in clinical interviews of patients by clinicians experienced in clinical dementia research. If a potential patient is found not capable of fully providing consent for participation, then his or her surrogate will provide consent and the patient will be asked to provide assent. Potential patients who are found to be able to provide informed consent will be asked to do so and their surrogates will co-sign the consent form as a witness. More details regarding the consent process can be found in the section on the protection of human subjects.

7.3. Enrollment visit

The enrollment visit includes an eligibility evaluation and a baseline component. At the start of the visit, study personnel will provide prospective patients with information about the study. After recruitment and consent, patients, with input from their caregivers, will undergo a comprehensive evaluation by a study physician and a study nurse to determine if they meet entry criteria. If entry criteria are satisfied, the prospective patient will start with the baseline assessments.

At the screening portion of the visit, study staff will:

- Discuss the study with the prospective patient and caregiver and obtain consent
- Assess and record eligibility
- Perform mental state exams
- Record patient and caregiver demographics
- Perform an electrocardiogram (ECG)

Once eligibility criteria are satisfied, study staff will:

- Obtain the name and address of the patient's personal physician, if any
- Perform physical and neurological exams
- Obtain a medical history and record current medications
- Obtain vital signs

- Collect a blood sample for DNA banking and electrolyte panels
- Collect patient assessments:
  - NeuroBehavioral Rating Scale (NBRS)
  - Modified AD Cooperative Study - Clinical Global Impression Worksheet (mADCS-CGI)
  - Cohen-Mansfield Agitation Inventory (CMAI)
  - Cornell Scale for Depression in Dementia (CSDD)
  - Neuropsychiatric Inventory (NPI)
  - AD Cooperative Study—Activities of Daily Living Scale (ADCS-ADL)
  - Mini Mental State Exam (MMSE)
  - Get up and Go (GUG)
- Provide the psychosocial intervention to the caregiver
- Obtain the randomized treatment assignment
- Dispense study medications and review instructions for medication use
- Review visit schedule, compliance monitoring, and adverse event reporting

As noted above, blood will be collected for the banking of DNA as a study resource. At the end of the study, genotyping using these banked specimens will be done to identify genetic polymorphisms associated with agitation in AD and response to agitation therapy.

8. Follow-up visits and telephone contacts

8.1. Overview of follow-up visits

Follow-up will include both scheduled and unscheduled visits and contacts. Scheduled follow-up includes:

- Scheduled in-person visits (weeks 3, 6, and 9 after randomization)
- Telephone contacts (weeks 1, 2, 4.5, and 7.5 after randomization)

Target dates for follow-up visits are calculated from the date of randomization.

8.2. Scheduled in-person visits

At all scheduled in-person visits, study staff will:

- Review study procedures to verify ongoing consent
- Review interval medical history
- Review and record current medications
- Obtain vital signs
- Collect blood for electrolyte panels and citalopram levels
- Perform an electrocardiogram (ECG) (at week 3 for all patients and an additional ECG at next scheduled visit for patients whose dose is increased to 3 capsules after week 3)
- Collect patient assessments:
  - NeuroBehavioral Rating Scale (NBRS)
  - Modified AD Cooperative Study - Clinical Global Impression of Change (mADCS-CGIC)
  - Cohen-Mansfield Agitation Inventory (CMAI)
  - Neuropsychiatric Inventory (NPI)
  - AD Cooperative Study—Activities of Daily Living Scale (ADCS-ADL)
  - Mini Mental State Exam (MMSE)
  - Get up and Go (GUG)
- Provide the psychosocial intervention to the caregiver
- Receive and record the amount of unused study drug
- Dispense a new supply of study drug (except week 9)
- Review visit schedule and compliance
- Discuss adverse events
- Refer the patient to a study physician, primary care physician, or specialist if the patient exhibits a notable change in condition or is medically unstable

8.3. Citalopram levels and electrolyte panels

Patients will be asked to provide blood to record citalopram levels at weeks 3, 6, and 9. These samples are obtained for assessing adherence and will be used to test the hypothesis that levels predict response. Patients also provide blood for electrolyte panels to be used for safety monitoring.

The dose of study medication, date and time of last dose of citalopram, and date and time of plasma sample obtained will be recorded. Citalopram plasma levels will be processed at the end of the trial to ensure that clinicians remain masked to the treatment assignment.

8.4. Telephone contacts

To ensure regular contact with study staff during the study, there will be telephone contact with patients and their caregivers at weeks 1, 2, 4.5, and 7.5 after randomization. The purpose of these contacts is to:

- Enhance compliance and retention
- Review visit schedule
- Provide medical monitoring
- Administer the agitation subitems of the NPI
- Collect adverse events

Medical information obtained from patients or caregivers is to be recorded for that contact.

8.5. Unscheduled follow-up visits and contacts

In addition to the visits outlined in the above schedule, patients may also be asked to appear for other assessments as needed. Unscheduled clinic visits may occur to evaluate a new or altered medical condition, to assess side effects, to assist with compliance in complex cases, or to provide counseling and behavioral interventions as needed.

Patients may contact the clinical site personnel between scheduled contacts regarding medical or cognitive problems that they are experiencing. Information will be recorded on the nature of the complaints and on any recommendation or referral made by clinical site personnel.

8.6. Data collection by visit

	EN	T1	T2	F3	T4	F6	T7	F9
Weeks from EN	0	1	2	3	4.5	6	7.5	9
**Procedures**								
Consent	✓	✓	✓	✓	✓	✓	✓	✓
History, or interim history	✓	.	.	✓	.	✓	.	✓
Vital signs, ongoing medical monitoring	✓	.	.	✓	.	✓	.	✓
Blood								
DNA	✓	.	.	.	.	.	.	.
Electrolyte panels	✓	.	.	✓	.	✓	.	✓
Citalopram levels	.	.	.	✓	.	✓	.	✓
Review of compliance	.	.	.	✓	.	✓	.	✓
Review of medication use	.	✓	✓	✓	✓	✓	✓	✓
Review inclusion/exclusion	✓	.	.	✓	.	✓	.	✓
Review of adverse events	.	✓	✓	✓	✓	✓	✓	✓
Dispensing of study drug	✓	.	.	✓	.	✓	.	.
Perform electrocardiogram (ECG)	✓	.	.	✓	.	✓*	.	✓*
**Patient assessments**								
NBRS	✓	.	.	✓	.	✓	.	✓
CGI	✓	.	.	✓	.	✓	.	✓
CMAI	✓	.	.	✓	.	✓	.	✓
CSDD	✓	.	.	.	.	.	.	.
NPI	✓	.	.	✓	.	✓	.	✓
NPI agitation domain	.	✓	✓	.	✓	.	✓	.
ADL	✓	.	.	✓	.	✓	.	✓
MMSE	✓	.	.	✓	.	✓	.	✓
GUG	✓	.	.	✓	.	✓	.	✓
**Caregiver intervention**								
Psychosocial intervention	✓	.	.	✓	.	✓	.	✓

*Assessment will be performed as needed. See section 8.2.

9. Outcomes assessment and neuropsychological testing

- *AD Cooperative Study—Activities of Daily Living Scale (ADCS-ADL)*[18]: This measure is an ADL inventory developed by the ADCS to assess functional performance in patients with AD. In a structured interview format, informants are queried as to whether subjects attempted each of 24 items in the inventory during the prior 4 weeks and their level of performance. The scale discriminates well among the stages of severity of AD, from very mild to severely impaired.

- *Cohen-Mansfield Agitation Inventory* (*CMAI*)[31]*: CMAI* is one of the most widely used agitation indicators for dementia-related clinical trials. It was developed by directly observing behavioral disturbances in dementia patients, and examines several agitated behaviors including verbal, physical agitation and other behaviors. Most dementia agitation trials have included CMAI. It has the advantage of being a good rating scale for both community and institutionalized dementia patients (Leaner 1997) both of whom will be represented in CitAD.

- *Cornell Scale for Depression in Dementia (CSDD)*[4]: This is a widely used measure of depression in the context of dementia, which has been shown to be sensitive to change in assessing treatment response in AD-associated depression. It involves a comprehensive approach to rate specific symptoms with input from patients and caregivers and exhibits a high concordance with the clinical diagnosis of major, minor, and absent depression.

- *Get up and Go (GUG)*[33]: Get Up and Go is a performance-based, sensitive measure of function in older persons. GUG measures the time a person takes to get up from a chair and walk 50ft (15.2m) as fast as possible along a level and unobstructed corridor. It has been used in clinical trials in which assessment of mobility is needed as a primary outcome, or as an indicator of adverse functional effects of treatments. GUG will be used in CitAD as a sensitive measure of drug effects on balance and gait stability.

- *Mini Mental State Exam (MMSE)*: This is a well-known cognitive screening test for the detection of dementia and estimation of its severity. The particular domains most often affected in AD (orientation, memory) are included in this instrument. Other items include those assessing working memory, naming, following verbal and written commands, spontaneously writing a sentence, and copying two overlapping pentagons.

- *Modified AD Cooperative Study - Clinical Global Impression of Change (mADCS-CGIC)*[50]: The mADCS-CGIC is a systematic method, developed for the AD setting to assess clinically significant change in a patient's agitation. A trained clinician, blind to treatment assignment, uses a 7-point Likert scale to rate each patient along a continuum from

"marked improvement " (1), "no change" (4), and "marked worsening" (7). Ratings will be based on an interview with the caregiver and examination of the patient. The mADCS-CGIC requires the assessor to consider a number of aspects of the agitation prior to providing a "global" assessment of change. These include: emotional agitation, mood liability/distress, psychomotor agitation, verbal aggression, physical aggression.

- *NeuroBehavioral Rating Scale (NBRS)*[23]: NBRS is a 28-item instrument rated by an observer and patient and derived from the Brief Psychiatric Rating Scale (BPRS) that assesses multiple types of psychopathology. It combines coverage of the breadth of psychopathology addressed in the BPRS with more comprehensive assessment of impairment seen in dementia. Scoring on the NBRS is based on a seven point Likert scale of increasing severity (i.e. 0=not present, 1=very mild, 2-mild, 3=moderate, 4=moderately severe, 5=severe, 6=extremely severe). Pollock (2002) used NBRS for agitation in dementia patients and reported that a first-order seven-factor model of the NBRS is the best fit for correlations among neurobehavioral rating scale items. Consequently, they used the seven NBRS items loading on the agitation factor to create an NBRS agitation domain score. In Pollock's previous trials, this agitation domain score was sensitive to drug treatment effects with citalopram. The agitation domain score includes NBRS items 8, 11 and 14, with a total range of 0 to 18 points.

- *Neuropsychiatric Inventory (NPI) sum of non-mood domains*[9]: The two domains for delusions and hallucinations will be used to assess severity of psychotic symptoms. The NPI caregiver distress scale will be used to evaluate caregiver distress caused by neuropsychiatric symptoms and agitation. Frequency (1=occasionally, less than once/week; 4 = very frequently, once or more/day or continuously) and severity (1=mild, 2=moderate, 3=severe) scales in each domain are scored based on responses from an informed caregiver involved in the patient's life. To obtain an NPI score for each domain, the severity score is multiplied by the frequency score.

Other measurements

- Blood pressure, pulse, respiratory rate, and weight will be measured using standard clinical procedures.

- Electrolyte panel will be assessed at enrollment and weeks 3, 6, and 9 post-randomization. The panel will include sodium, potassium, chloride, bicarbonate, glucose, urea nitrogen, creatinine and magnesium levels.

- Adverse events and serious adverse events will be defined using standard approaches. The occurrence of adverse events and serious adverse events will be noted by the site clinical teams and recorded in the study documents for analytic purposes.

10. Analysis plan

10.1. General principles

General principles for analysis include the following:

- The primary analysis will be performed according to the patients' original treatment assignment (intention to treat), regardless of administered treatment
- All patients, including those who are found to be ineligible after randomization or those who withdraw from the study, will be counted in their assigned treatment group once the treatment assignment has been revealed
- All events following randomization will be counted
- Multiple imputation will be used to impute outcomes for those subjects with missing measures

Analyses will be done to look for differences in the outcome between the treatment groups. Results of these analyses will be presented unadjusted and adjusted for covariates. Covariates to be used for adjusting treatment group effects will include clinical site (the stratification variable) and other prospective baseline risk factors chosen with clinical judgment and/or variable selection procedures such as forward selection. Exploratory analyses will be performed in which post randomization data, such as adherence to the assigned treatment regimen or treatment received, will be taken into account. In addition, treatment effects will be examined across various subgroups, including sites. However, power to detect subgroup differences will be limited, so these analyses also are exploratory.

Formal procedures will not be used for adjusting p-values resulting from multiple treatment group comparisons. With respect to interim monitoring, it is to be expected that numerous comparisons of treatment efficacy and safety must be performed over the course of a clinical trial. Rather than to adjust p-values for multiple comparisons, p-values will be interpreted as descriptive statistics of the evidence, and not as absolute indicators for a positive or negative result.

10.2. Analysis of primary outcomes

Neuro-Behavioral Rating Scale (NBRS) [Primary outcome]. NBRS will be measured with in-person interviews at baseline (week 0) and at weeks 3, 6, and 9. To capitalize on the longitudinal design, the outcomes will be treated as repeated measures. It is hypothesized that patients on citalopram will have lower NBRS scores over the course of 9 weeks compared to those on placebo. The analysis will be performed using the standard repeated measures Multivariate Analysis of Covariance (MANCOVA) for multiple visits as summarized below (for details on such models, see Laird and Ware, 1982; and Johnson and Wichern, 1992)[21,19]. In the basic model of this analysis, for subject I the outcome $y_{i,t}$ at time t=3, 6, or 9 is assumed to be $y_{i,t} = a_t + b_t * y_{i,0} + D_t * G_i + e_{i,t}$, where $G_i = 0$ for subjects on placebo and $G_i = 1$ for subjects on treatment; $y_{i,0}$ is the outcome at baseline (treated as a covariate because it is measured before randomized treatments start); a_t and b_t are to be estimated so that $a_t + b_t * y_{i,0}$ is the average outcome at time t for subjects on placebo and with

baseline outcome equal to $y_{i,0}$; D_t is the average difference between treatment and placebo outcomes at time t conditional on the baseline measure of the outcome. Also, conditional on treatment arm and baseline outcome, $e_{i,t}$ for t=3, 6, 9, are normal random variables with means 0 and are allowed to have arbitrary variances and correlations among each other, which allows for different variability of the outcomes at different times, and for dependence between the outcomes of the same subject at different times. The model we will use for the analysis will be the above basic model where, to also adjust for possible center effects, in the above equation we will also include and estimate terms $F_{center(I)}$ where center(I) indicates the center at which patient (I) is randomized. Fitting and estimation of this model will be done using the maximum likelihood estimation method with the statistical package R. In this approach, the hypothesis of superiority of treatment to placebo translates to having the differences $D_t < 0$ for $t > 0$. An overall assessment of treatment effect will be evaluated by estimating the average $D_{all} = (D_3 + D_6 + D_9)/3$, and testing the null hypothesis that $D_{all} = 0$.

mADCS-CGIC [Primary outcome]. The categorical outcome on the mADCS-CGI of each patient at week 9, which compares each patient's overall agitation outcome at endpoint to that at the baseline visit on a seven point scale (1 = marked improvement, 2 = moderate improvement, 3 = minimal improvement, 4 = no change, 5 = minimal worsening, 6 = moderate worsening, 7 = marked worsening) will be compared by randomization group. It is hypothesized that the proportion of patients with worse scores on CGI scale is lower on citalopram than placebo. To formulate this hypothesis statistically, consider the proportions, $p_{y,G=0}$, of patients at category y, among those on placebo, and the proportions $p_{y,G=1}$ of patients at category y, among those on citalopram. Also, consider the cumulative proportions $q_{y,G=0}$ of patients at or worse than category y, among those assigned placebo, and those proportion $q_{y,G=1}$ of patients at or worse than category y, among those assigned citalopram. The research hypothesis then can be translated in that the odds ratios $R_y = \{q_{y,G=1} /(1- q_{y,G=1})\} / \{q_{y,G=0} /(1- q_{y,G=0})\}$, for treatment versus placebo of patients being at or worse than category y, are lower than 1. To capitalize on the ordered categories, the hypothesis will be evaluated with the proportional odds method[34]. This method assumes that the odds ratios R_y are constant across the y categories, i.e. $R_y = R$. Although this assumption may not necessarily be exactly correct, this method has the correct 0.05 type I error under the null hypothesis H_0 of no treatment effect because, under H_0, the proportional odds assumption is satisfied exactly with an odds ratio of 1 between treatment groups across all outcome categories. Moreover, this method is known to be considerably more powerful than unstructured methods for detecting ordered alternative hypotheses to H_0, such as our research hypothesis, even when these alternatives do not exactly satisfy the proportional odds assumption[34]. Maximum likelihood estimates using this method will be calculated with the statistical package R using the procedure POLR[55].

10.3. Analysis of secondary outcomes

Cohen-Mansfield Agitation Inventory (CMAI), NPI agitation subitem ratings, and cumulative lorazepam dose [secondary outcomes]. CMAI ratings are done every three weeks and NPI-agitation subitem ratings are obtained weekly for the first 3 weeks and then approximately every 10 days until 9 weeks, either by phone or in-person. Total dose of lorazepam used will also be recorded. It is hypothesized that patients on citalopram will have lower CMAI and NPI agitation ratings over 9 weeks, and lower cumulative lorazepam dose compared to those on placebo.

CMAI ratings will be analyzed using the same methods as NBRS scores described in the previous section. NPI agitation subitem telephone ratings will be analyzed, and the results will be compared to those of NPI in-person agitation ratings. If no significant difference is found, NPI agitation subitem ratings from both types of interviews will be combined and examined (secondary analyses, with linear mixed effects models) to narrow focus on possible critical time points, where the trajectories of the citalopram group may start diverging from those of the placebo group. Total lorazepan salvage dose will be compared between randomized groups with the Wilcoxon test.

Patient outcomes will include ratings at baseline and weeks 3, 6, and 9 on the NPI-delusions and hallucinations domains, AD Cooperative Study-Activities of Daily Living scale (ADCS-ADL), Mini Mental State Exam (MMSE), and caregiver total distress with NPS ratings on the NPI. It is hypothesized that patients on citalopram will have better outcomes over the course of 9 weeks compared to those on placebo. Most of these measures are continuous longitudinal outcomes and will be analyzed as described for the primary objective. Categorical longitudinal outcomes (i.e., emergence of delusions or hallucinations) will be compared between randomized groups using a multinomial logistic model fitting the marginal distributions across time and randomized group, which will be estimated with GEE[24] and an AR-1 working autocorrelation matrix.

It is also hypothesized that citalopram will be as well tolerated as placebo for vital signs, weight, gait stability, and side effect measured at baseline and at weeks 3, 6, and 9. For continuous outcomes, the model is as for NBRS, but the analysis plan has the following modifications. First, in the possible case where citalopram effects can be relatively smaller in earlier compared to later weeks, the comparison $D_{all} = (D3 + D6 + D9)/3$ may inappropriately dilute a later difference. Therefore, since for safety it is most important to have accurate probability of finding a difference if one exists, we will assess safety by comparing D9, at week 9. Second, since this is an equivalence hypothesis, a one-sided test will be used, as recommended by Piantadosi[40]. For categorical outcomes, the model and estimation will be as described for modeling NPI delusions and hallucinations, with the modification that we will only model baseline and week 9, to avoid possible false claims of equivalence due to dilution.

We will also examine predictors of agitation reduction and of response to citalopram by looking at plasma levels of citalopram in a population pharmacokinetic study, genetic predictors, and baseline clinical characteristics. It is hypothesized that these characteristics can explain part of the variation in

agitation reduction and response to citalopram. The above characteristics will be examined as predictors in the MANCOVA model as described in the primary objective, in the proportional odds model as described in the primary objective, or in the marginal categorical model as described in the secondary objective, depending on the type of outcome.

Adverse events and discontinuation of study treatment. We hypothesize that patients randomized to citalopram will develop significantly more adverse events than patients treated with placebo, but that rates of discontinuation due to adverse events will be similar in the two groups. The proportion of patients who experience adverse events and who discontinue double blind treatment will be compared at each follow-up between randomized arms using logistic regression.

All measures will be evaluated for outliers, and distributional assumptions will be checked to ensure applicability of the statistical procedures. For subjects with missing outcomes, multiple imputation will be used. Every effort will be made to minimize noncompliance in the trial. With noncompliance, intention-to-treat analysis is unbiased for the effect of randomization, but not for the effect of taking the treatment. So in addition to the primary intention-to-treat analysis, treatment effect may evaluated in secondary analyses with two other methods:

- “As-treated" analysis, which compares subjects by the received treatment, adjusting for observed baseline prognostic factors. This is a standard exploratory method, but it too can be biased for the effect of taking the treatment if the subjects who take treatment have different unmeasured prognostic factors independent of treatment after adjusting for the measured prognostic factors.

- Instrumental variables analysis. Under certain plausible conditions, these types of analyses are practically unbiased for the effect of taking the treatment even if there are unmeasured prognostic factors between subjects who do and do not take the treatment. Moreover, these methods better account for non-constant treatment effects and incomplete observations[17].

11. Data monitoring

The monitoring of accumulating data from CitAD on safety and efficacy will be done by an independent data and safety monitoring board (DSMB). The DSMB members are not involved in the conduct of CitAD and are free of affiliations with the manufacturer of citalopram. The DSMB will be convened by phone in the first year of the study to review its role, procedures, and practices. It will meet at least once a year thereafter, in-person or by telephone, to review safety reports and the conduct of the study. Up to three non-voting study representatives, designated by the director of the CC, will participate in the meetings.

Monitoring reports of the accumulating data presented to the DSMB will include treatment group comparisons of baseline characteristics, measures of disturbance, changes over time in measures of functioning and cognition, and adverse events. DSMB members will not be masked to treatment assignment. The board may recommend that the trial should be stopped before its planned conclusion if they observe convincing evidence of a treatment difference in agitation outcomes or safety issues.

12. Quality assurance and performance monitoring

12.1. Overview

Quality assurance strategies for CitAD include design strategies and monitoring activities. Design strategies include use of randomization to assign patients to treatment groups, masking data collectors to treatment assignment to the extent possible, requirement of certification of staff and sites, and formal training of staff in study procedures. Activities to monitor quality include performance monitoring, visits to clinical sites, and error detection procedures.

12.2. Certification of sites

Study investigators will be required to complete a clinical site certification form that provides detailed information with regard to the space, facilities, and personnel at the site. One purpose of the form is to serve as a checklist for staff of the resources that need to be in place when participant activities begin. Additional items requested will be a copy of the IRB notice of approval and copies of the stamped consent statements to be used at the site. The information provided will be reviewed by Coordinating Center staff prior to certification of a clinical site for data collection.

12.3. Training of staff

Personnel from the Chairman's Office and Coordinating Center will train the physicians, coordinators, nurses, and psychometricians in the standardized and uniform use of all assessment instruments prior to the randomization of patients in the trial. Training methods will include didactic instruction and clinical demonstrations. Training materials documenting the counseling strategies employed in the caregiver intervention will also be used. The nurses who administer the psychosocial intervention will be trained in its application. This training will occur at the start of the study and will be updated annually. As appropriate, standardized methods for performing study procedures will be outlined in the handbook.

12.4. Certification of staff

The purpose of the staff certification program is threefold. It identifies to the Coordinating Center and to the study group and to the staff who will collect and/or record certain items of data for CitAD and who will make decisions relating to eligibility. Second, it makes the data collector aware that he/she is a part of CitAD and has a responsible and identifiable role in the trial. Third, it helps to document a minimum level of competency to perform the functions of the staff person's role.

Personnel to be certified for CitAD include study physicians, study nurse/coordinators, psychometricians, and data system operators. Staff will be reminded of their duties and responsibilities to the participants, each other, and the public in adhering to high ethical standards in their interactions with participants and each other, in protecting the privacy of trial participants and the confidentiality of their records, in collecting accurate and reliable data, and in adhering to principles for the analysis and reporting of the data. They will be reminded of their duty to make known to proper authorities any suspicious or wrongful actions in relation to these processes. Each

member of the research group will be asked to sign a statement indicating a knowledge and understanding of the above and to voluntarily disclose any potential conflicts of interest. The next best set of assurances lies in the use of design strategies that protect the results from treatment-related biases. Strategies proposed for this trial include random assignment of patients to active treatment or placebo and masked data collection and outcome assessment. Beyond these strategies, assurances depend on the documents, methods, and procedures used for data collection and monitoring.

12.5. Performance monitoring

Performance monitoring will begin with the initiation of participant screening and will continue throughout the duration of the trial. Sites will be monitored on a regular basis regarding the following:

- Rate of enrollment
- Protocol deviations
- Missed visits
- Losses to follow-up
- Completeness of data
- Percentage of data items requiring edit queries
- Percentage of discrepancies found in audited data items
- Timing of visits

Summaries of the above measures will be provided to the sites and to the DSMB on a regular basis. Review of performance data will be an agenda item for the annual investigators meetings.

12.6. Site visiting

Site visits will be made to each of the clinical sites early in the course of recruitment and at other points in time as needed or desired for quality assurance purposes. The site visitors will review consent forms for enrolled participants, study documents, IRB approvals, staffing, adverse event reports, protocol issues, forms management, data management, and study drug accounting records.

12.7. Error detection

The study will employ double data entry and range checks to reduce the occurrence of errors. In addition, some logic checks will be done at the CC, and these checks may be updated throughout the trial to address new data problems as they are discovered. Edit queries will be made to the clinical sites on a regular basis regarding inconsistencies. Periodically, additional batch edits related to consistency of data across forms and over time will be generated.

Periodic audits of subsets of the database will be conducted, both through visits to the sites and through a remote auditing procedure. At on-site visits, participant data will be chosen for verification from source documentation. For the remote auditing procedure, the CC will periodically review participant form sets.

13. Protection of human subjects

13.1. Human subjects

Participants will be adults, who meet clinical and research criteria for dementia of the Alzheimer's type, and their caregivers. There will be no restrictions to participation based on gender, race, ethnicity, or age. We expect to enroll participants representative of the ethnic and racial diversity of the geographic and patient populations of the participating institutions. Entry criteria are intended to be as inclusive as possible in order to obtain a clinical sample typical of the patients likely to receive study treatments in the usual clinical situation.

13.2. Monitoring of IRB approvals

One of the requirements for certification of a site to begin participant activities will be submission to the Coordinating Center of the site's notice of IRB approval and a copy of each stamped consent form used at the site. These materials will be reviewed by Coordinating Center staff for inclusion of appropriate elements.

Sites that have obtained IRB approval for a previous version of the protocol will inform their IRB of changes to the protocol. Protocol amendments and changes to the consent form will be distributed from the Coordinating Center via numbered memos. These amendments and changes will be submitted by the sites to their IRB in writing.

Reporting local and study-wide adverse events will be done according to each local IRB's policy. The IRBs also will receive a summary of study-wide adverse event data (not by treatment assignment) on an annual basis.

13.3. Consent procedures

Patients (or their legal proxies) and caregivers who may potentially fulfill study criteria will be approached by clinic or research staff to assess initial interest. If the patient and caregiver are interested, study personnel are to explain the study to them and obtain voluntary written informed consents required for participation in this study. This will be obtained from patients with Alzheimer's disease (or authorized surrogates) and from the patient's caregiver who will be providing data on themselves as a caregiver. No study procedures will be undertaken or study medications administered until such consents are obtained. Even after a patient has provided initial consent to participate, we will use enrollment and subsequent visits, and the implementation of study procedures as opportunities to again explain what is being done and to assure continuing informed consent on the part of the proxy, to maintain assent by the patients, and to assess capacity. Patients and their authorized legal representatives will be included and consented at the study sites using local procedures established by the individual sites and their overseeing IRBs in accordance with local law.

In all cases, prospective patients with dementia will first be assessed for their ability to provide informed consent. Capacity to give consent will be assessed in clinical interviews of patients by clinicians experienced in clinical dementia research who will also be trained in obtaining consent for the study. In the course of these interviews, these clinicians will assess the ability of patients to:

- Comprehend the study and its consent form, by asking them to repeat the key elements of the research
- Understand the study and its consent form, by answering questions about the key elements of the research
- Appreciate the personal nature and consequences of what will or could happen to them should they agree to participate

Interviews will take place in the presence of a person who may act as a surrogate research decision maker, if needed. The designation of a surrogate will be governed by local state and IRB rules. In general, this will be, in order of priority: a legal guardian, someone who holds a research advance directive for the patient, a healthcare agent by advance directive, or a healthcare surrogate decision maker by local law or custom, such as a spouse, adult child, or sibling.

If in this process, a potential patient is found not capable of fully providing consent for participation, then the surrogate will provide consent in their place and the patient will be asked to provide assent. The process of obtaining consent and assent will be documented in every case. If potential patients are able to provide informed consent, they will be asked to do so and their surrogates will co-sign the consent form as a witness. Caregivers will only be individuals who can provide informed consent for themselves and will be asked to provide informed consent for participation as informants and also to provide data on themselves as caregivers in the course of the study.

13.4. Consent procedures for DNA blood collection

Any patient who participates in CitAD will also be asked to consent to blood collection for DNA banking. Blood collection for DNA banking is optional: Patients can be enrolled in the study even if they choose not to provide a blood sample. Patients may withdraw their consent to participate in genotyping or other DNA studies at any time without affecting their ability to participate in CitAD.

13.5. Potential risks and benefits

Potential risks

The major risks of this study involve the potential adverse effects of citalopram and the risk of being assigned to placebo, thus prolonging discomfort and suffering. However, it is not currently knowable in advance whether citalopram is efficacious for a particular patient. There is a risk that an individual may be initially assigned to placebo and may not improve, or his or her agitation may worsen as a result. However, these risks will be mitigated by the use of a psychosocial intervention, salvage lorazepam, and the close monitoring of patients. The risk of being in the study with respect to

receiving medications is comparable to receiving medication in ordinary clinical settings. The optimal duration of treatment with an antidepressant medication for agitation is not known. Some patients will be treated with medication for up to 9 weeks. It may later be discovered, as a result of the analysis of this trial or others, that this length of time is too long for some patients, and may have resulted in some unanticipated side effects. Nevertheless, the overall evaluation, clinical care, monitoring, and medication administration are likely to be more intensive and careful than usual clinical care, and to this extent participation in this trial may be associated with less risk.

The antidepressant chosen for this study, citalopram, has been on the market for several years, has been used extensively with elders, and is generally well tolerated. Initial data about side effects in Alzheimer's patients come from the Pollock trial[41]. The most common side effects are gastrointestinal upset, nausea, vomiting, diarrhea, tremors, headaches, or restlessness. Less common are delirium, falls, confusion, weight loss, agitation, or sexual dysfunction, and rarely hyponatremia or "serotonin syndrome". There are also risks associated with the use of lorazepam. These include sedation, confusion, gait instability, falls, and very rarely—although not usually seen with short term use—addiction. In August 2011 the FDA announced that citalopram was associated with a dose dependent QT prolongation and should not be used at doses over 40 ms per day.[15] The data the FDA used to support the announcement did not include any patients over 50 years of age (Thomas Laughren (FDA), personal communication). The increase in the QT interval at 40 ms was estimated to be 12.6 ms with 90% confidence intervals ranging from 10.9 to 14.3 ms. There is of course the potential for unexpected side effects including death in this frail population of patients, as the recent meta-analysis for atypical antipsychotics suggests[48]. However the risks of unexpected side effects are minimal given the extensive pre- and post-marketing world-wide experience with citalopram in millions of people, including millions of elderly people.

Potential benefits

Patients will be closely monitored throughout their participation in the study. Failure to achieve meaningful clinical improvement will result in changes in treatment designed to maximize the likelihood of such improvement. The benefits to society of this study will include important new data on the treatment of agitation occurring with dementia.

Risk/benefit ratio

Risks to the patients from the study medication will be similar to those encountered by other elderly patients with dementia who receive treatment with these drugs. Risks to the patients from lack of treatment efficacy will be minimized by the several regular assessments and by the investigators' abilities to discontinue or change medicines or to treat patients openly if they are not benefitting from their current protocol-specified treatment. The benefits that participants will receive in the form of free and systematic treatment, in addition to the societal benefits of important treatment data concerning a disorder with substantial morbidity and mortality, outweigh the risks to participating subjects. The projected outcomes following participation in this trial are similar to what the participants may expect in the absence of participation, except that treatment is likely to be

monitored more carefully, and optimized. Therefore, the anticipated net benefits from participation are likely to be at least as great as those to be expected in the absence of participation.

Protection of subjects and close monitoring

The medication to be used in this study has been evaluated and approved by the FDA for clinical use for the treatment of depression. Although there is no currently available indication for the treatment of agitation of Alzheimer's dementia, this medication is in fact used extensively by clinicians for treating psychiatric complications of dementia. The risks and benefits of citalopram, of specific-study procedures, and of the study as a whole will be explained in detail to patients, caregivers, and responsible parties. After a medical and psychiatric history, patients will undergo physical, neurological, and psychiatric examinations to assure the clinical appropriateness and safety of their participation. Close clinical monitoring will ensure the appropriateness and safety of their continued participation. Close monitoring will include several components: a) careful education of caregivers to monitor patients at home, including provision of written materials on how to contact the team and what side effects to look for (in lay language); b) weekly telephone contact during the medication titration phase followed by in-clinic contact every 3 weeks with telephone contacts between in-person visits; c) contact with an experienced nurse, at minimum; d) in-clinic visits with a prescribing physician; and e) availability of the study team by 24-hour beeper for assistance with crises and urgent or emergent matters and for personal support.

13.6. Safety monitoring

Study personnel will have frequent contact with participants both by phone and in-person. Patients will be monitored regularly for signs or symptoms of adverse effects. In addition, the DSMB will regularly review and evaluate accumulating safety data and may recommend termination of the trial if the risks become unacceptable.

13.7. Confidentiality of patient and caregiver data

Clinical sites will keep all patient and caregiver data in a secure location. Names, addresses, and other such personal data will not be part of the central database. Data collected from study evaluations and interviews will be identified only by study ID codes, which will be the patient ID number and 4-letter code assigned at eligibility evaluation. Caregivers also will be identified by an ID code. Any disclosure of potentially identifiable health information will be done in accordance with the law.

14. Biohazards

Blood will be collected for DNA banking, electrolyte panels, and citalopram levels. All personnel involved in collecting and handling biologic specimens are to follow appropriate precautionary procedures as currently recommended by the Centers for Disease Control and Prevention. Shipping of specimens are to be done in compliance with federal regulations.

15. Literature cited

1. Aalten P, de Vugt ME, Jaspers N, Jolles J, Verhey FR. The course of neuropsychiatric symptoms in dementia. Part I: findings from the two-year longitudinal Maasbed study. Int J Geriatr Psychiatry. 2005 Jun;20(6):523-30.

2. Aalten P, de Vugt ME, Jaspers N, Jolles J, Verhey FR. The course of neuropsychiatric symptoms in dementia. Part II: relationships among behavioral sub-syndromes and the influence of clinical variables. Int J Geriatr Psychiatry. 2005 Jun;20(6):531-6.

3. Alexopoulos GS, Katz IR, Reynolds CF, Carpenter D, Docherty J. Pharmacotherapy of depressive disorders in older patients. Postgraduate Medicine October 2001, McGaw Hill.

4. American Psychiatric Association. Practice Guideline for the treatment of patients with Alzheimer's disease and other dementias of late life. Am J Psychiatry 1997; 154: (Supplement 5): 1-39.

5. Beck AT, Ward CH, Mendelson M, Mock J, Erbaugh J. An inventory for measuring depression. Arch Gen Psychiatry 1961; 4: 561-571.

6. Blair JR, Spreen O. Predicting premorbid IQ: A revision of the National Adult Reading Test. The Clinical Neuropsychologist 1989; 3:129-136.

7. Borkowski JG, Benton AL, Spreen O. Word fluency and brain damage. Neuropsychologia 1967; 5:135-140.

8. Brookmeyer R, Gray S, Kawas C.: Projections of Alzheimer's disease in the United States and the public health impact of delaying disease onset. Am J Public Health 1998 Sep;88(9):1337-42

9. Cummings JL, Mega M, Gray K, Rosenberg-Thompson S, Carusi DA, Gornbein J. The Neuropsychiatric Inventory: Comprehensive Assessment of Psychopathology in Dementia. Neurology 1994; 44: 2308-2314.

10. Davis KL, Marin DB, Kane R, Patrick D, Peskind ER, Raskind MA, Puder KL. The Caregiver Activity Survey (CAS): Development and validation of a new measure for caregivers of persons with Alzheimer's disease. International Journal of Geriatric Psychiatry. 1997;12(10): 978-88.

11. Doody RS, Stevens JC, Beck C, et al. Practice parameter: Management of dementia (an evidence based review). Report of the Quality Standards Subcommittee of the American Academy of Neurology. Neurology 2001; 56:1154-1166.

12. Ferri CP, Prince M, Brayne C, et al. Global prevalence of dementia: a Delphi consensus study. Lancet. 2005; 366(9503):2112-7.

13. Fillit HM. The pharmacoeconomics of Alzheimer's disease. Am J Manag Care 2000 Dec;6(22 Suppl):S1139-44; discussion S1145-8.

14. Finkel SI, Mintzer JE, Dysken M, Krishnan KR, Burt T, McRae T. A randomized, placebo-controlled study of the efficacy and safety of sertraline in the treatment of the behavioral manifestations of Alzheimer's disease in outpatients treated with donepezil. Int J Geriatr Psychiatry. 2004 Jan;19(1):9-18.

15. Food and Drug Administration website. (22 August 2011): http://www.fda.gov/Drugs/DrugSafety/ucm269086.htm

16. Fox PJ, Kohatsu N, Max W, Arnsberger P. Estimating the costs of caring for people with Alzheimer disease in California: 2000-2040. J Public Health Policy 2001;22(1):88-97.

17. Frangakis CE, Rubin DB, Zhou XH. Clustered encouragement design with individual non-compliance: Bayesian inference and application to Advance Directive Forms. Biostatistics 2002 (with Discussion); 3: 147-164.

18. Galasko D, Bennett D, Sano M, Ernesto C, Thomas R, Grundman M, Ferris S. An inventory to assess activities of daily living for clinical trials in Alzheimer's disease. The Alzheimer's Disease Cooperative Study. Alzheimer Disease & Associated Disorders, 1997; 11(Suppl 2): S33-9.

19. Johnson R A, Wichern DW. Applied multivariate statistical analysis. London: Prentice-Hall, 1987.

20. Kirby D, Ames D. Hyponatraemia and selective serotonin re-uptake inhibitors in elderly patients. Int J Geriatr Psychiatry. 2001 May;16(5):484-93.

21. Laird NM and Ware JH. (1982). Random-effects models for longitudinal data. Biometrics, 38, 963-974.

22. Laughren T. A regulatory perspective on psychiatric syndromes in Alzheimer's disease. Am J Geriatr Psychiatry 2001; 9:340-345.

23. Levin HS, High WM, Goethe KE, et al. The Neurobehavioral Rating Scale: assessment of the behavioral sequale of head injury by the clinician. J Neurol Neurosurg Psychiatry 1987; 50: 183-193

24. Liang, KY and Zeger, SL (1986), Longitudinal data analysis using generalized linear models. Biometrika 73: 13--22.

25. Lingjaerde O, Ahlfors UG, Bech P, et al, The UKU Side Effect Rating Scale: a new comprehensive rating scale for psychotropic drugs and cross-sectional study of side effects in neuroleptic treated patients. Acta Psychiatr Scand Suppl 1987; 334: 1-100

26. Livingston G, Johnson K, Katona C et al. Systematic review of psychological approches to the management of neuropsychiatric symptoms of dementia. Am J Psychiatry 2005; 162: 1996-2021

27. Lyketsos, Constantine G, Brookmeyer R, Gray S, Kawas C.: Projections of Alzheimer's disease in the United States and the public health impact of delaying disease onset. Am J Public Health 1998 Sep;88(9):1337-42

28. Lyketsos CG. Neuropsychiatric Symptoms (a.k.a. BPSD) and dementia treatment development. International Psychogeriatrics, Published online by Cambridge University Press 09Mar2007; doi:10.1017 /S104161020700484X.

29. Lyketsos CG, Colenda C, Beck C, Blank K, Doriaswamy M, Kalunian D, Yaffe K. Position statement of the American Association for Geriatric Psychiatry regarding principles of care for patients with dementia due to Alzheimer's disease. Am J Geriatr Psychiatry 2006;14(7):561-72.

30. Magai C, Kennedy G, Cohen CI, Gomberg D. A controlled clinical trial of sertraline in the treatment of depression in nursing home patients with late-stage Alzheimer's disease. Am J Geriatr Psychiatry. 2000 Winter;8(1):66-74.

31. Cohen-Mansfield J, Billig N. Agitated behaviors in the elderly. I. a conceptual review. J Am Geriatrics Soc 1986; 34(10): 711-21.

32. Martin BK, Frangakis CE, Rosenberg PB, Mintzer JE, Katz IR, Porsteinsson AP, Schneider LS, Rabin PV, Munro CA, Meinert CL, Niederehe G, Lyketsos CG, for the DIADS-2 Research Group. Design of Depression in Alzheimer's Disease Study-2. Am J Geriatr Psychiatry 2006;14(11):920-930.

33. Mathias S, Nayak U S L, Isaacs B. Balance in the elderly: The “get-up and go” test. Arch Phys Med Rehab 1986;67:387–9.

34. McCullagh P. Regression models for ordinal data. Journal of the Royal Statistical Society, Ser B, 1980; 42: 109-142.

35. Munro CA, Brandt J, Sheppard JME, Steele CD, Samus QM, Steinberg M, Rabins PV, Lyketsos CG. Cognitive response to pharmacological treatment for depression in Alzheimer disease: secondary outcomes from DIADS. Am J Ger Psychiatry 2004; 12: 491-498.

36. Nelson, H.E.. The National Adult Reading Test (NART): Test manual. Windsor, Berkshire, UK: NFER-Nelson, 1982.

37. Nyth AL, Gottfries CG: The clinical efficacy of citalopram in treatment of emotional disturbances in dementia disorders: A Nordic multicentre study. Brit J Psychiatry 1990; 157:894-901.

38. Nyth AL; Gottfries CG; Lyby K; Smedegaard-Andersen L; Gylding-Sabroe J; Kristensen M; Refsum HE; Ofsti E; Eriksson S; Syversen S 1992 A controlled multicenter clinical study of citalopram and placebo in elderly depressed patients with and without concomitant dementia Acta Psychiatr Scand 1992 Aug;86(2):138-45.

39. Petracca G, Tesón A, Chemerinski E, Leiguarda R, Starkstein SE. A double-blind placebo-controlled study of clomipramine in depressed patients with Alzheimer's disease. J Neuropsychiatry Clin Neurosci 1996 Summer;8(3):270-5.

40. Piantadosi S. (1997). Clinical Trials: a methodologic perspective. New York: Wiley.

41. Pollock BG, Mulsant BH, Rosen J, Sweet RA, Mazumdar S, Bharucha A, Marin R, Jacob NJ, Huber KA, Kastango KB, Chew ML. Comparison of citalopram, perphenazine, and placebo for the acute treatment of psychosis and behavioral disturbances in hospitalized, demented patients. Am J Psychiatry. 2002 Mar;159(3):460-5.

42. Rabins PV, Lyketsos CG, Steele CD. Practical Dementia Care. Oxford University Press, New York, 2006.

43. Rabins PV, Lyketsos CG. Antipsychotic drugs in dementia: what should be made of the risks? JAMA 2005; 294:1963-1965

44. Reitan RM, Wolfson, D. The Halstead-Reitan Neuropsychological Test Battery: Theory and clinical interpretation. Tucson, AZ: Neuropsychology Press, 1993.

45. Rosen WG, Mohs RC, Davis KL. A new rating scale for Alzheimer's disease. American Journal of Psychiatry 1984; 141(11): 1356-1364.

46. Ryu SH, Katona C, Rive B, Livingston G. Persistence of and changes in neuropsychiatric symptoms in Alzheimer's disease over 6 months - the LASER-AD Study, American J Ger Psychiatry 2005; 13 (11) 976-983.

47. Schneider LS, Tariot PN, Dagerman KS, Davis SM, Hsiao JK, Ismail MS, Lebowitz BD, Lyketsos CG, Ryan JM, Stroup TS, Sultzer DL, Weintraub D, Lieberman JA; CATIE-AD Study Group. Effectiveness of atypical antipsychotic drugs in patients with Alzheimer's disease. N Engl J Med 2006 Oct 12;355 (15):1525-38.

48. Schneider LS, Dagerman KS, Insel P. Risk of death with atypical antipsychotic drug treatment for dementia: metaanalysis of randomized placebo-controlled trials. JAMA. 2005 Oct 19;294(15):1934-43.

49. Schneider LS, Nelson JC, Clary CM, Newhouse P, Krishnan KR, Shiovitz T, Weihs K; Sertraline Elderly Depression Study Group. An 8-week multicenter, parallel-group, double-blind, placebo-controlled study of sertraline in elderly outpatients with major depression. Am J Psychiatry. 2003 Jul;160(7):1277-85

50. Schneider LS, Olin JT, Doody RS, Clark CM, Morris JC, Reisberg B, Schmitt FA, Grundman M, Thomas RG, Ferris SH. Validity and reliability of the Alzheimer's disease Cooperative Study-Clinical Global Impression of Change. The Alzheimer's disease Cooperative Study. Alzheimer disease & Associated Disorders 1997; 11(Suppl 2):S22-32.

51. Stern Y, Albert SM, Sano M, Richards M, Miller L, Holstein M, Albert M, Bylsma FW, Lafleche G. Assessing patient dependence in Alzheimer's disease. J Gerontol. 1994; 49(5): M216-22.

52. Steinberg M, Munro CA, Rabins PV, Brandt J, Lyketsos CG. Patient predictors of response to treatment of depression in Alzheimer's disease: The DIADS study. Int J Geriatr Psychiatry 2004; 19:144-150.

53. Steinberg M, Shao H, Zandi P, Tschanz J, Welsh-Bohmer KA, Norton M, Breitner JCS, Steffens D, Lyketsos CG. Point and Five-year Period Prevalence of Neuropsychiatric Symptoms in Dementia: The Cache County Study. Poster at the International Conference on Alzheimer's disease bi annual meeting, Madrid, July 2006.

54. Tollefson GD, Sayler ME. Course of psychomotor agitation during pharmacotherapy of depression: analysis from double-blind controlled trials with fluoxetine. Depress Anxiety. 1996-1997;4(6):294-311.

55. Venables WN, Ripley BD. Modern applied statistics with S-Plus. (3rd ed) New York: Springer-Verlag, 2000.

56. Wechsler D. Wechsler Memory Scale-Third Edition. Administration and Scoring Manual. New York: The Psychological Corporation, 1997.

57. Wolfson C, Wolfson DB, Asgharian M, M'Lan CE, Ostbye T, Rockwood K, Hogan DB for the Clinical Progression of Dementia Study Group. NEJM 2001; 344:1111-6.

Appendix 1: Power calculations

Power calculations were conducted for two hypotheses: citalopram is better than placebo on NBRS through week 9; and citalopram is better than placebo on mADCS-CGIC. For the second hypothesis, sample size is determined by standard two sample methods. For the first hypothesis, important information exists from pilot studies, so determination of sample size involved three components: a range of values for a clinically meaningful difference was specified; a simulation model was used to translate the clinically meaningful difference and estimates from relevant previous studies (pilots) into expected distributions of the data; and the analysis models specified in the 'Analysis plan' section were used to calculate the power to detect differences in the simulated distributions. The simulation component is important because the analysis models estimate more of the distributions' underlying structure than simply the clinical difference. The simulation model then uses the estimates from the pilot studies and the clinical difference to predict the likely underlying structure that the analysis model will face, and which determines power. All power calculations are for the intention-to-treat distributions, i.e., they already reflect the distributions of the outcomes in the groups assigned to placebo and sertraline regardless of compliance.

For the first hypothesis, the outcome was the difference in average NBRS scores at week 9 between the randomized groups; the following pilot information was derived from the preliminary studies (B. Pollock, personal communication): the mean (=10.08) and standard deviation (= 4.81) of $y_{i,0}$ when assigned citalopram, the mean (=8.81) and standard deviation (=5.63) of $y_{i,6weeks}$ when assigned citalopram, and the standard deviation (=4.58) of the change $y_{i,6weeks} - y_{i,0}$ when assigned citalopram; and the predicted ratio (=3.5%) of within person variance to total variance at week 6 among patients assigned citalopram. This pilot information and the desired clinical difference at week 9 were used to determine the parameters of a linear mixed effects model with random intercept and random slope (Laird and Ware, 1982), with which we predicted the likely covariance structure and the expected averages at baseline and at weeks 3, 6, and 9 for each randomized group. Based on this structure, the below table gives the total sample size required for different values of power and effect size. For comparison, we also give the sample size needed to achieve the same power with the t-test.

Total sample size needed by power and effect size variation for NBRS by week 9. Plain numbers are for longitudinal analyses, and numbers in parentheses are for a t-test at week 9			
Power	75%	80%	85%
Effect size			
40%	148	172	196
	(174)	(196)	(224)
50%	96	110	124
	(112)	(126)	(144)
Sample size is for a scenario with full data. The sample size for anticipating m% attrition can be obtained by dividing the given sample size by (1-m/100)			

In the second hypothesis, the power of the preferred analysis described in the 'Analysis plan' section is not exactly known because we do not have available pilot data on this outcome for such patients. Nevertheless, it is expected based on antipsychotic agitation in AD trials (Schneider, 2006) that 20%-30% of patients assigned to placebo will improve considerably compared to baseline (score at least 2 by week 9), in which case we wish to have enough power to detect a difference of 20-30% between citalopram and placebo in the proportions of patients who improve (or worsen). The below table gives sample size for such scenarios, for power based on the two-sample comparison of proportions (Piantadosi, 1997, ch 7.4.3). These sample sizes can be taken as upper bounds of the sample sizes required with the more powerful analysis based on the proportional odds model described in the 'Analysis plan' section.

**Total sample size, power and proportions of patients improving in the mADCS-CGIC scale**			
**Power**	**75%**	**80%**	**85%**
**p_0=20%**			
**Effect 20%**	144	160	186
**25%**	96	106	124
**30%**	68	84	88
**p_0=25%**			
**Effect 20%**	156	176	202
**25%**	102	114	132
**30%**	72	88	94
**p_0=30%**			
**Effect 20%**	166	186	212
**25%**	108	120	138
**30%**	76	88	96

p_0: proportion of patients assigned placebo with mADCS-CGIC at least 2 by week 9.
Effect: difference between citalopram and placebo arms of the proportion with mADCS-CGIC at least 2 by week 9.
Sample size is for a scenario with full data. The sample size for anticipating m% attrition can be obtained by dividing the given sample size by (1-m/100).

Appendix 2: CitAD design summary

Title

- Citalopram for Agitation in Alzheimer's Disease (CitAD)

Objectives

- Primary objective
  - To examine in a masked, randomized trial the efficacy of citalopram for the treatment of clinically significant agitation, without major depression, in patients with Alzheimer's dementia

- Secondary objectives
  - To examine the effects of citalopram treatment on function and cognition of the patient as well as caregiver burden
  - To examine the safety of citalopram for the treatment of clinically significant agitation, without depression, in patients with AD by comparing the citalopram and placebo treatment groups on the following: vital signs, weight, gait stability, side effects, electrolyte panels, and adverse event reports
  - To examine predictors of response to citalopram therapy

Type of trial

- Randomized, multicenter clinical trial
- Two parallel treatment groups
- Double masked
- 1:1 assignment ratio

Design variable

- Agitation measured by NBRS

Primary outcome measures

- Agitation over 9 weeks as measured by NBRS
  - It is hypothesized that patients on citalopram will have lower NBRS scores over the course of 9 weeks compared to those on placebo
- Change in agitation as measured by mADCS-CGIC
  - It is hypothesized that the proportion of patients with worse scores on CGI scale is lower on citalopram than placebo

Other outcomes

- Agitation over 9 weeks as measured by CMAI
- Agitation over 9 weeks as measured by NPI agitation sub-items
- Need for rescue medication for agitation as measured by cumulative lorazepam dose
  - It is hypothesized that patients on citalopram will have lower CMAI and NPI agitation ratings over nine weeks, and lower cumulative lorazepam dose compared to placebo
- Functional performance as assessed by ADCS-ADL
  - It is hypothesized that patients on citalopram will have better outcomes on functional performance assessment measured by ADL
- Cognition as assessed by MMSE
- Caregiver distress as assessed by caregiver distress ratings on NPI
  - Adverse events and serious adverse events
  - It is hypothesized that citalopram will be as well as tolerated as placebo for vital signs, weight, and gait stability

Study population

- 200 patients who meet the CitAD criteria for clinically significant agitation

Sample size and power calculations

- Two-sided alpha = 0.05
- Power greater than 85%
- 40% reduction in agitation severity as measured by NBRS

Treatments

- Citalopram, target dose 30 mg per day (range 10-30 mg per day), given orally + standardized psychosocial intervention
- Placebo + standardized psychosocial intervention

Stratification

- By clinical center

Masking

- Double-masked (treatment assignment masked to participants and all personnel, including physicians, nurses, and neuropsychologists)

Inclusion criteria

- Probable Alzheimer's disease (NINCDS-ADRDA criteria), with MMSE score of 5-28 inclusive
- A medication for agitation is appropriate, in the opinion of the study physician
- Clinically significant agitation for which either
    1) the frequency of agitation as assessed by the NPI is 'Very frequently', or
    2) the frequency of agitation as assessed by the NPI is 'Frequently' AND the severity of the agitation as assessed by the NPI is 'Moderate', or 'Marked'
- Provision of informed consent for participation in the study by patient or surrogate (if necessary) and caregiver
- Availability of primary caregiver, who spends several hours a week with the patient and supervises his/her care, to accompany the patient to study visits and to participate in the study
- No change to AD medications within the month preceding randomization, including starting, stopping, or dosage modifications

Exclusion criteria

- Meets criteria for Major Depressive Episode by DSM-IV (TR) criteria
- Presence of a brain disease that might otherwise explain the presence of dementia, such as extensive brain vascular disease, Parkinson's disease, dementia with Lewy bodies, traumatic brain injury, or multiple sclerosis
- Psychosis (delusions or hallucinations) *requiring antipsychotic treatment* in the opinion of the study physician
- Prolonged QT interval
- Treatment with citalopram is contraindicated in the opinion of the study physician
- Failure of past treatment with citalopram for agitation after adequate trial at a minimally accepted dose (≥20 mg/day)
- Treatment with a medication that would prohibit the safe concurrent use of citalopram, such as MAO inhibitors
- Need for acute psychiatric hospitalization or suicidal
- Current participation in a clinical trial or in any study that may add a significant burden or affect neuropsychological or other study outcomes
- Current treatment with antipsychotics, anticonvulsants (other than dilantin), other antidepressants (other than trazodone, ≤50 mg per day at bedtime), benzodiazepines (other than lorazepam), or psychostimulants
- Any condition that, in the opinion of the study physician, makes it medically inappropriate or risky for the patient to enroll in the trial

---

Duration of follow-up

- 9 weeks

Data collection schedule

- Scheduled in-person visits (weeks 3, 6, and 9 after randomization)
- Telephone contacts (weeks 1, 2, 4.5, and 7.5 after randomization)
