# Supplementary material for: Changes in QTc Interval in the Citalopram for Agitation in Alzheimer's Disease (CitAD) Randomized Trial
Source: PLoS One. 2014 Jun 10;9(6):e98426. doi: 10.1371/journal.pone.0098426 (PMC4051660; doi:10.1371/journal.pone.0098426)
Supplement: Text S1 — Sensitivity analyses supporting information. (DOCX) [file pone.0098426.s004.docx]

## S1 Sensitivity analyses supporting information

One participant in the placebo group had a very low enrollment QTc and large change in QTc (see Figure 3) compared to other participants and as a result, this participant’s data were influential in analyses. The unusual data were not due to data transcription or entry error; the study cardiologist (DS) manually re-read the ECG tracing and thought that the computer estimate of the baseline QTc could be an underestimate. Therefore, we also performed analyses excluding this influential outlier. After exclusion, the difference in week 3 QTc adjusting for baseline QTc was: 20.7 ms [95% CI: 9.7, 31.7; p = 0.0005]. For both of the binary outcomes (increase > 30ms and prolonged QTc), the previous analyses counted the highly influential placebo participant as the one event in the placebo group; after excluding this participant the p-value for the difference between the groups was more extreme (for increase greater than 30ms, p = 0.009; for prolonged QTc, p = 0.110). The slope estimate of the change in QTc by baseline QTc was not different than a slope of zero (indicating no relationship) after excluding the outlier (slope estimate = -0.175 [95% CI: -0.480, 0.130]; p = 0.253).
